# Restricting sugar or carbohydrate intake does not impact physical activity level or energy intake over 24 h despite changes in substrate use: a randomised crossover study in healthy men and women

**DOI:** 10.1007/s00394-022-03048-x

**Published:** 2022-11-03

**Authors:** Aaron Hengist, Russell G. Davies, Peter J. Rogers, Jeff M. Brunstrom, Luc J. C. van Loon, Jean-Philippe Walhin, Dylan Thompson, Françoise Koumanov, James A. Betts, Javier T. Gonzalez

**Affiliations:** 1 Department for Health, University of Bath, Bath, BA2 7AY UK; 2 Centre for Nutrition, Exercise and Metabolism, University of Bath, Bath, UK; 3 School of Psychological Sciences, University of Bristol, Bristol, UK; 4 Department of Human Biology, Maastricht University, Maastricht, The Netherlands

**Keywords:** Ketogenic, Metabolism, Energy balance, Exercise

## Abstract

**Purpose:**

To determine the effects of dietary sugar or carbohydrate restriction on physical activity energy expenditure, energy intake, and physiological outcomes across 24 h.

**Methods:**

In a randomized, open-label crossover design, twenty-five healthy men (*n* = 10) and women (*n* = 15) consumed three diets over a 24-h period: moderate carbohydrate and sugar content (MODSUG = 50% carbohydrate [20% sugars], 15% protein, 35% fat); low sugar content (LOWSUG = 50% carbohydrate [< 5% sugars], 15% protein, 35% fat); and low carbohydrate content (LOWCHO = 8% carbohydrate [< 5% sugars], 15% protein, 77% fat). Postprandial metabolic responses to a prescribed breakfast (20% EI) were monitored under laboratory conditions before an ad libitum test lunch, with subsequent diet and physical activity monitoring under free-living conditions until blood sample collection the following morning.

**Results:**

The MODSUG, LOWSUG and LOWCHO diets resulted in similar mean [95%CI] rates of both physical activity energy expenditure (771 [624, 919] vs. 677 [565, 789] vs. 802 [614, 991] kcal·d^−1^; *p* = 0.29] and energy intake (2071 [1794, 2347] vs. 2195 [1918, 2473] vs. 2194 [1890, 2498] kcal·d^−1^; *P* = 0.34), respectively. The LOWCHO condition elicited the lowest glycaemic and insulinaemic responses to breakfast (*P* < 0.01) but the highest 24-h increase in LDL-cholesterol concentrations (*P* < 0.001), with no differences between the MODSUG and LOWSUG treatments. Leptin concentrations decreased over 24-h of consuming LOWCHO relative to LOWSUG (*p* < 0.01).

**Conclusion:**

When energy density is controlled for, restricting either sugar or total dietary carbohydrate does not modulate physical activity level or energy intake over a 24-h period (~ 19-h free-living) despite substantial metabolic changes.

**Clinical trials registration ID:**

NCT03509610, https://clinicaltrials.gov/show/NCT03509610

**Supplementary Information:**

The online version contains supplementary material available at 10.1007/s00394-022-03048-x.

## Introduction

Carbohydrates are a dietary staple for many people, providing ~ 50% of energy intake in high-income countries [1–3]. When carbohydrate intake is restricted to the extent where endogenous carbohydrate stores become substantially depleted, liver-derived ketone bodies provide an alternative fuel for the brain and skeletal muscle [4, 5]. Achieving ketosis through dietary carbohydrate restriction is known as a ketogenic diet. Ketogenic diets have become popular as a method of reducing body mass, and there is current discourse on the mechanisms by which carbohydrate restriction may alter body mass or composition [6, 7]. Ultimately, to influence long-term body mass and composition, any nutritional intervention must influence at least one component of energy balance. Of the components of energy balance, one important but often neglected component is physical activity energy expenditure, which is causally affected by nutrition to magnitudes relevant for body composition [8, 9].

An alternative approach to restricting all carbohydrates for altering energy balance and body composition is to specifically restrict sugar intake. Indeed, meta-analysis reveals higher sugar intakes (~ 12% increase) increase energy intake by ~ 265 kcal/d [10], particularly when added sugars are ingested in sugar-sweetened beverages—a major source of sugar intake in high-income countries [11, 12]. Therefore, public health guidelines advocate restriction of added or free sugars, typically to less than 5% of total energy intake [10, 13], despite some limitations with the quality of the evidence [14, 15]. Manipulating dietary sugars, however, results in modest (< 1 kg) changes in body mass, and isoenergetic exchange of free sugars with other sources of carbohydrates does not change body mass [16]. The relatively small decreases in body mass seen with sugar restriction, when taken in concert with the more substantial reduction in energy intake, suggest that other components of energy balance (e.g., physical activity) may be compensating to erode the energy deficit. To the best of the authors knowledge, there is to date, no causal data on the role of dietary sugars on all components of energy balance, especially those most likely to respond to such an intervention like physical activity.

Prior evidence indicates potential for carbohydrate availability to alter physical activity. Alternate day fasting can decrease physical activity by > 100 kcal⋅d^−1^ [17] and men and women randomized to morning fasting display ~ 440 kcal⋅d^−1^ lower physical activity than those consuming a carbohydrate-rich breakfast [8]. These dietary stimuli have an immediate impact on physical activity—without the need for weeks of intervention to manifest—but it remains unclear if these effects are specific to restriction of total energy, carbohydrate, or sugar per se. Indeed, evidence from rodents indicates a direct role of hepatic glycogen stores in regulating physical activity [18], which implicates dietary sugars on the basis that fructose potently stimulates hepatic glycogenesis [19]. Only one study has estimated physical activity in response to ketogenic carbohydrate restriction in lean adults (*n* = 5), demonstrating a 20% reduction in indirectly estimated physical activity with a low-carbohydrate, ketogenic diet versus a high-carbohydrate diet [20]. Therefore, to the best of the authors knowledge, no studies to date, have directly measured the effects of ketogenic carbohydrate, or sugar restriction on physical activity energy expenditure. Accordingly, the present study investigated whether the quantity of carbohydrate and/or sugar consumed alters 24-h physical activity energy expenditure in humans. In addition, we established the acute metabolic responses to these diets and their effects on appetite and energy intake. We hypothesized that carbohydrate restriction would reduce 24-h physical activity energy expenditure compared to higher-sugar and/or higher-carbohydrate intake.

## Methods

### Study design and sample size

**Fig. 1:**
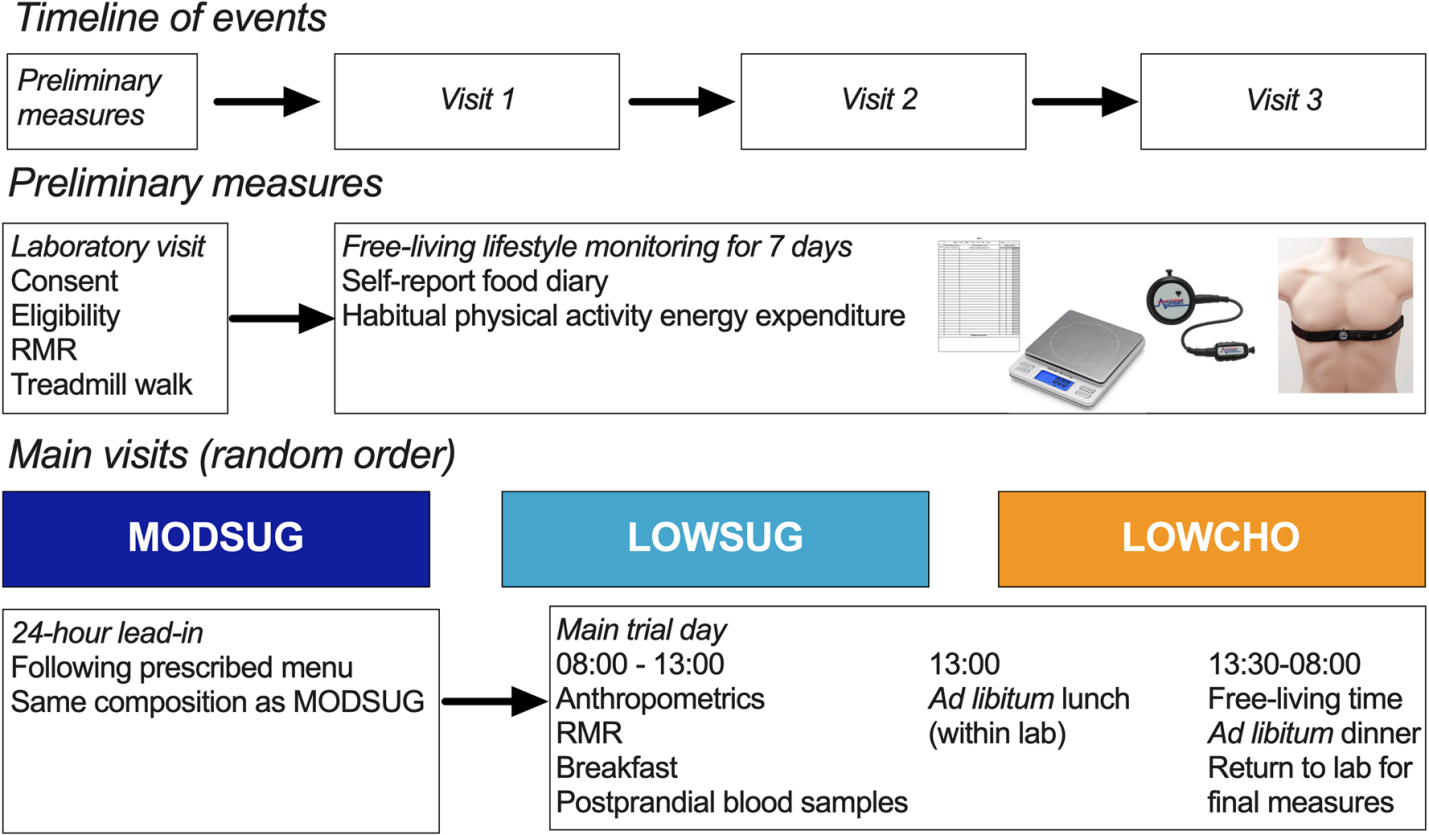
Schematic of study design. RMR, resting metabolic rate; MODSUG, moderate-sugar diet; LOWSUG, low-sugar diet; LOWCHO, low-carbohydrate diet

Twenty-five men and women (Table 1) participated in an open-label (participants blinded to primary outcome but not intervention), randomized, crossover design. Participants completed 1 week of habitual diet and physical activity monitoring, before completing 3 laboratory visits in a random order, during which they standardised diet for 24-h prior, then received breakfast in the laboratory with the 4-h postprandial responses measured, before they received an ad libitum lunch. Participants left the laboratory for the remainder of the day immediately following lunch, were provided with ad libitum dinner to consume outside of the laboratory, and returned the following morning. No restrictions were placed on free-living physical activity outside the laboratory, and participants did not know this was the primary outcome as confirmed using an exit questionnaire during the final visit. A schematic of the study design is presented in Fig. 1. The study was approved by the Research Ethics Approval Committee for Health at the University of Bath (EP 17/18 78) and all measures were conducted in accordance with the Declaration of Helsinki with participants providing written informed consent. The study was registered at clinicaltrials.gov (NCT03509610). Trial order randomization was completed using randomizer.org. Inclusion criteria were body mass index 18.5–29.9 kg∙m^−2^, age 18–65 years, and no anticipated changes in diet and physical activity during the study (e.g., holidays or diet plans). Exclusion criteria were any reported condition or behaviour that might pose undue personal risk or introduce bias, diagnosed metabolic disease (e.g. type 2 diabetes), lifestyle not conforming to standard sleep–wake cycle (e.g. shift worker), and any reported change in body mass greater than 3% in the previous 6 months [21].

**Table 1:** Participant characteristics at time of preliminary measures

	Female (*n* = 15)	Male (*n* = 10)	Combined (*n* = 25)
Age (years)	26 ± 7 (18–43)	26 ± 9 (19–48)	26 ± 8
Height (m)	1.68 ± 0.05 (1.55–1.76)	1.79 ± 0.04 (1.72–1.83)	1.72 ± 0.07
Body mass (kg)	62.3 ± 6.0 (52.9–72.4)	75.2 ± 10.8 (59.2–92.1)	67.5 ± 10.3
Body fat (%)	26.8 ± 4.6 (15.6–32.3)	16.4 ± 4.9 (9.6–25.0)	22.6 ± 7.0
Body mass index (kg m^−2^)	22.1 ± 1.8 (19.4–25.9)	23.6 ± 3.2 (20.0–29.8)	22.7 ± 2.5
Waist circumference (cm)	73.6 ± 4.3 (65.0–82.0)	84.0 ± 10.1 (72.3–99.5)	77.8 ± 8.8
Hip circumference (cm)	98.4 ± 4.7 (92.5–106.0)	99.5 ± 5.7 (92.5–110.0)	98.8 ± 5.1
Waist: hip (ratio)	0.75 ± 0.04 (0.68–0.82)	0.84 ± 0.06 (0.76–0.95)	0.79 ± 0.07
Habitual energy intake (kcal·d^−1^)	2137 ± 387 (1603–2826)	2702 ± 412 (2177–3453)	2280 ± 465
Habitual carbohydrate intake (g·d^−1^)	236 ± 51 (185–337)	299 ± 81 (188–425)	261 ± 70
Habitual carbohydrate intake (g·kg·d^−1^)	3.8 ± 0.7 (3.0–5.5)	4.1 ± 1.3 (2.2–6.0)	3.9 ± 1.0
Habitual sugars intake (g·d^−1^)	104 ± 27 (68–157)	125 ± 48 (66–227)	112 ± 38
Habitual sugars intake (g·kg·d^−1^)	1.7 ± 0.4 (0.9–1.9)	1.7 ± 0.8 (0.7–3.2)	1.7 ± 0.6
Habitual fat intake (g·d^−1^)	88 ± 18 (52–114)	107 ± 23 (63–149)	95 ± 22
Habitual fat intake (g·kg·d^−1^)	1.4 ± 0.3 (0.9–1.9)	1.5 ± 0.4 (0.7–2.1)	1.4 ± 0.4
Habitual protein intake (g·d^−1^)	78 ± 18 (48–110)	112 ± 19 (79–139)	92 ± 24
Habitual protein intake (g·kg·d^−1^)	1.3 ± 0.3 (0.7–1.8)	1.5 ± 0.2 (1.1–1.9)	1.4 ± 0.3
Habitual alcohol intake (g·d^−1^)	11 ± 10 (0–32)	12 ± 10 (0–33)	11 ± 10
Habitual alcohol intake (g·kg·d^−1^)	0.2 ± 0.2 (0.0–0.6)	0.1 ± 0.1 (0.0–0.4)	0.2 ± 0.2
Habitual PAEE (kcal·d^−1^)*	946 ± 409 (389–1687)	1623 ± 435 (1009–2149)	1171 ± 520

Data presented are mean ± SD (min–max)

*Female *n* = 10, male *n* = 5

The required sample size was estimated based on the Bath Breakfast Project [8] using G*Power 3.1 software [22]. Mean ± SD physical activity energy expenditure (PAEE) for the fasting vs. breakfast groups during the morning (when differences in carbohydrate availability between groups were present) were 311 ± 124 kcal vs. 492 ± 227 kcal. Based on this effect size (*d* = 0.998), a two-tailed matched-pairs design with 15 participants would provide > 90% chance (power) of detecting the stated effect with an α-level of 0.05. Due to a technical failure, data loss occurred for the first 10 participants for the primary outcome measure. As stated in the original protocol, a rolling recruitment to achieve the sample size of 15 for primary outcome measure (24-h physical activity energy expenditure) was continued which resulted in *n* = 15 for the primary outcome measure but a total sample of *n* = 25. The study team were unable to cannulate one participant due to small vasculature so blood samples could not be obtained. The higher sample size than initially planned enabled exploratory analyses of sex differences in response to the different diets.

### Diets

**Fig. 2:**
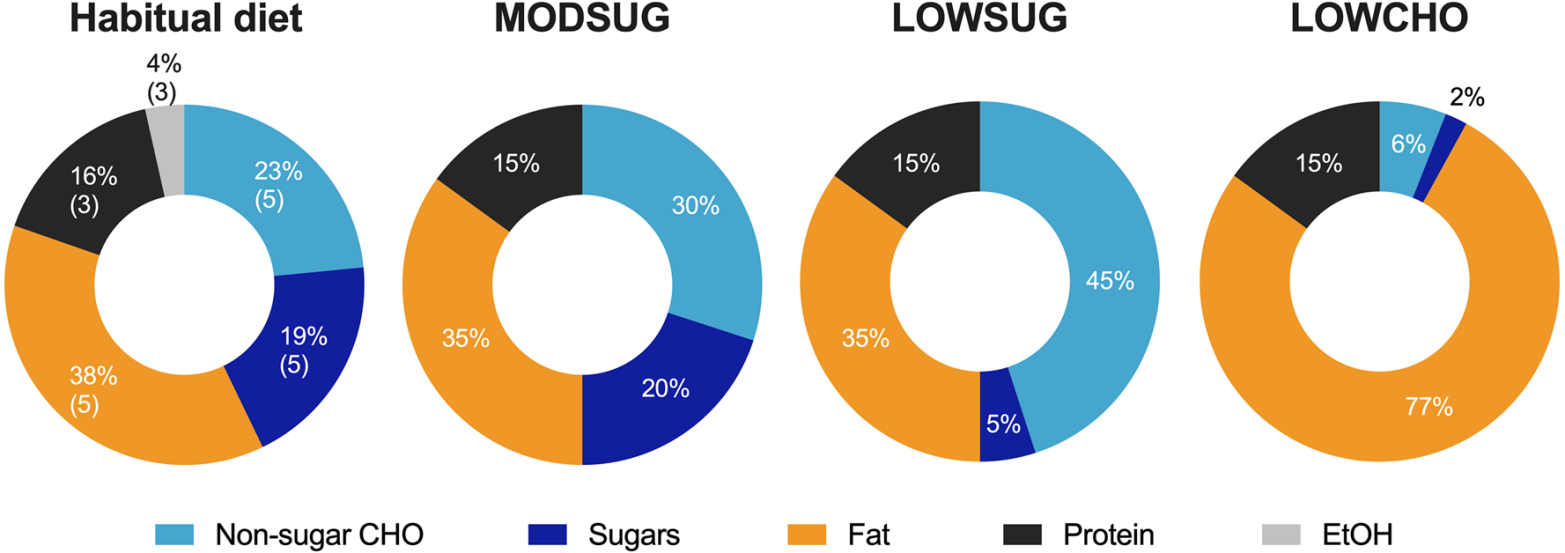
Dietary macronutrient intake as a percentage of total energy intake for habitual diet and each of the three experimental conditions. *MODSUG* moderate-sugar diet, *LOWSUG* low-sugar diet, *LOWCHO* low-carbohydrate diet, *CHO* carbohydrates, *EtOH*, alcohol. For habitual mean and SD are shown

We investigated 3 diets (Fig. 2). The moderate-sugar (MODSUG) diet was designed to be reflective of macronutrient and sugar intake in European populations [1, 23], the low sugar (LOWSUG) diet was designed to meet UK public health guidelines that advocate reducing free sugar intake to < 5% of total energy intake [10, 24], and the low-carbohydrate (LOWCHO) diet was designed to restrict carbohydrate availability and promote ketogenesis, consistent with the definition of a ‘very low-carbohydrate ketogenic diet’ [25]. Estimated caloric values for each nutrient were used to calculate energy intake: carbohydrates 3.75 kcal g^−1^, sucrose 3.94 kcal g^−1^, fat 8.94 kcal g^−1^, protein 4.02 kcal g^−1^, and alcohol 6.93 kcal g^−1^ [26]. The UK labelling system currently requires the reporting of ‘total sugars’ rather than ‘free sugars’ [27], therefore whilst we have aimed to manipulate free sugars between the diets, it can only assumed that most of the sugars in the present study are free sugars, so we refer to ‘sugars’ throughout. A description and nutritional information of the breakfast meals given to participants to consume in full within the laboratory is provided in Table 2. A description and nutritional information of the lunch and dinner meals given to participants to consume ad libitum within the laboratory (lunch) and outside of the laboratory (dinner) is provided in Table 3. Photographs of meals provided to participants are shown in Supplemental Fig. 1. The energy content of the breakfast meal was calculated on the first laboratory visit and replicated in subsequent trials. We aimed to provide 20% of total energy requirements as a typically-representative breakfast intake [28] and factor in confinement to the laboratory during the testing phase by using measured resting metabolic rate (RMR) and measured habitual physical activity energy expenditure (PAEE). To achieve this, we estimated energy requirements by combining 8 h of resting metabolic rate (RMR) for sleep, 6 h of RMR for the laboratory component of the trial (14 h total), and combined resting and physical activity energy expenditure (PAEE) for the remainder of waking hours (10 h) using the following equation:

1 $${\text{Total}}\,{\text{energy}}\,{\text{requirements}}\,\left( {{\text{kcal}}} \right) = \left[ {\left( {\frac{{{\text{RMR}}\left( {{\text{kcal}}} \right)}}{24}} \right) \times 14} \right] + \left\{ {\left[ {\left( {\frac{{{\text{RMR}}\left( {{\text{kcal}}} \right)}}{24}} \right) + \left( {\frac{{{\text{PAEE}}\left( {{\text{kcal}}} \right)}}{16}} \right)} \right] \times 100} \right\}.$$

**Table 2:** Description and nutritional information of each breakfast meal

	MODSUG	LOWSUG	LOWCHO
Description	Wholemeal toast with peanut butter, chocolate spread and grape juice	White and wholemeal toast with butter spread and water	Crushed avocado with chia seeds, scrambled egg and water
Energy density (kcal g^−1^)	1.65	1.65	1.59
Carbohydrate energy (%)	52	52	8
Fat energy (%)	33	33	76
Protein energy (%)	15	15	16
Sugars energy (%)	22	4	1
Fibre (g per 100 g)	2.9	3.1	2.9
Saturated fat (g per 100 g)	1.2	1.4	2.8
Monounsaturated fat (g per 100 g)	3.0	1.6	6.5
Polyunsaturated fat (g per 100 g)	1.6	2.9	2.1
Ratio (saturated/unsaturated)	0.26	0.32	0.32

**Table 3:** Description and nutritional information of each lunch/dinner meal

	MODSUG	LOWSUG	LOWCHO
Description	Pesto pasta with cheese and added sugar	Pesto pasta with cheese	Cauliflower and cheese with added pesto
Energy density (kcal g^−1^)	2.43 (0.08)	2.12 (0.09)	2.52 (0.04)
Carbohydrate energy (%)	52	52	3
Fat energy (%)	33	33	81
Protein energy (%)	15	15	16
Sugars energy (%)	22	2	2
Fibre (g per 100 g)	1.3 (0.0)	1.7 (0.3)	1.3 (0.0)
Saturated fat (g per 100 g)	3.3 (0.1)	2.8 (0.4)	7.9 (0.1)
Monounsaturated fat (g per 100 g)	3.0 (0.1)	2.4 (0.4)	7.4 (0.1)
Polyunsaturated fat (g per 100 g)	2.6 (0.1)	2.3 (0.3)	7.0 (0.1)
Ratio (saturated/unsaturated)	0.59	0.59	0.55

Data shown are mean (SD)

Lunch and dinner were ad libitum with 2000 kcal prepared for each meal. All lunch and dinner meals were prepared the evening before the trial day and refrigerated overnight and all bread was refrigerated but not frozen, as these practices influence resistant starch production and glycaemic responses to the ingested carbohydrates [29, 30]. Palatability of the breakfast and lunch meals was assessed by asking participants to strike a line through a 0–100 mm scale (0 = bad, 100 = good) shortly following ingestion of the meal. A palatability score was calculated by combining mean scores for ‘Visual Appeal’, ‘Smell’, ‘Taste’, and ‘Palatability’.

### Preliminary measures

Participants attended the laboratory for eligibility screening and a treadmill walk to calibrate the physical activity monitors. Then, participants completed 7 days of habitual lifestyle monitoring, which comprised a weighed food diary and wearing a combined accelerometer and heart rate monitor to measure free-living physical activity energy expenditure (Actiheart 4™, CamNtech Ltd., UK). Participants were provided with weighing scales to weigh food items (SmartWeigh, China) and food diaries were analysed using diet analysis software (Nutritics, Ireland).

### Laboratory visit standardization

Participants chose a menu aligned to the macronutrient intake of the MODSUG diet and were provided with food, weighing scales, and a physical activity monitor for 24 h leading into trial days. Participants were asked to record actual intake for 24 h leading into the first trial and replicate before this each subsequent trial, which was confirmed by writing on a printed menu. Participants were also asked to refrain from strenuous physical activity in the 24 h before each laboratory visit. Median (interquartile range) time between main visits was 13 (7–21) days for males and females using oral contraception, and 28 (28–34) days for menstruating females.

### Laboratory visits

Participants arrived at the laboratory following an overnight fast (duration 11:38 ± 00:57 hh:mm). They were asked to consume a glass of water and take an inactive transport mode rather than walk or cycle to the laboratory. Anthropometric measures of height, body mass, waist circumference, and hip circumference were obtained, and body fat percentage was estimated using digital scales (Tanita, Japan). Resting metabolic rate was measured using the Douglas bag technique by averaging three 5-min gas samples, with guidelines for best practice followed [31]. Expired fractions of O_2_ and CO_2_ were determined via paramagnetic and infrared analysers (Mini HF 5200, Servomex Group Ltd., UK), respectively, and the volume expired was measured using a dry gas meter (Harvard Apparatus, UK). Inspired O_2_ and CO_2_ were measured concurrently to account for ambient fluctuations [32]. Energy expenditure and substrate oxidation in the postprandial period was calculated using stoichiometric equations [33, 34], assuming urinary nitrogen excretion was negligible.

A cannula (BD Venflon™ Pro, Becton Dickenson & Co., Sweden) was inserted into a hand vein or antecubital forearm vein if hand cannulation was unsuitable and the arm was placed in a heated box (University of Vermont, USA) set to 55 °C to arterialize venous blood [35]. Participants completed a computer-based food preference task, which consisted of 18 plates of food individually photographed on a white plate or transparent bowl. Two foods were placed side-by-side and the participant selected which food they would ‘choose to eat right now’. Foods were distinguished into three categories: sweet high-carbohydrate foods, non-sweet high-carbohydrate foods, non-sweet low-carbohydrate foods which were matched at six levels for energy density and content. The task determines relative preference for these 3 food categories at each measurement. Participants completed 0–100 mm visual analogue scales to measure appetite; marking a line through the scale relating to how hungry, full, or thirsty they were and how strong their desire for sweet, savoury, rich, or creamy food with 0 corresponding to ‘not at all’ and 100 corresponding to ‘extremely’. A baseline blood sample was obtained, and the cannula was flushed with 10 mL sterile NaCl 0.9% (B. Braun, Pennsylvania, USA) to maintain patency throughout the trial (repeated at each blood sample). Participants were provided with breakfast at 09:12 ± 00:19 hh:mm and asked to ingest the whole meal within 15 min. A timer was started upon ingestion of the first bite of breakfast and metabolic responses were measured for 4 h. Blood samples were taken at 15-min intervals for the first hour and then every 30 min thereafter. A five-minute expired gas samples was collected within the final 10 min of each hour. Visual analogue scales were repeated hourly. Immediately following the 4-h postprandial period, participants completed a second computer task for food preferences with the hand still being heated. Following this the cannula was removed and participants were served with the lunch meal. Participants remained in the bed whilst bowls of the lunch meal were served to them in ~ 500 kcal portions. They were asked to eat until they were comfortably full. Food was served at 52.8 ± 3.7 °C (mean ± SD). Bowls were replaced at random time intervals with the aim that participants did not finish a portion and so could not estimate the quantity consumed.

Participants left the laboratory following lunch and returned the following morning using a similar mode of transport. Food was provided ad libitum between laboratory visits with no constraints on free-living energy expenditure. Participants were instructed to eat only the food provided and to drink only water for the rest of the day. Upon arrival the following morning, participants completed a visual analogue scale, a food preference task, and a 5-mL arterialized venous blood sample was obtained from an antecubital vein.

### Physical activity energy expenditure

Physical activity energy expenditure was measured using branched-equation modelling of heart rate and accelerometry across 24 h. Physical activity monitors were individually calibrated for each participant by using a treadmill protocol modified from Brage et al. [36]; participants attended the laboratory following a minimum 5 h fast and walked at a speed of 5.2 km h^−1^ for 20 min on a treadmill with incremental 5-min stages at gradients 0, 3, 6 and 9%. Expired gas samples were obtained in the final minute of each stage and analysed via indirect calorimetry to measure energy expenditure. Heart rate was obtained during the final minute of each stage using a chest-worn monitor (Polar Electro, Finland). Sleeping heart rate was measured during the 7 days of preliminary wear time. Mean resting metabolic rate from laboratory visits was entered as energy expenditure at sleeping heart rate. A linear model was fitted for energy expenditure at a range of heart rates from sleeping heart rate to the final stage of the treadmill walk and was extrapolated beyond this point for higher intensity activity. Thresholds for physical activity intensities were defined and calculated for each participant as sedentary < 1.5 METs, light ≥ 1.5–< 3.0 METs, moderate ≥ 3.0–< 6.0 METs, vigorous ≥ 6.0–< 10.2 METs, and very vigorous ≥ 10.2 METs [37, 38].

### Blood sampling and analyses

Blood samples were collected into tubes containing clotting activator (Sarstedt, Germany) and left at room temperature for 15 min before being centrifuged at 4000 × g for 10 min at 4 °C. Serum was aliquoted in duplicate into sterile tubes, placed on dry ice, and stored at − 80 °C. Serum glucose, triglycerides (TAG), glycerol, non-esterified fatty acids (NEFA), lactate, beta-hydroxybutyrate (βOHB), total cholesterol, high-density lipoprotein cholesterol (HDL-c), and low-density lipoprotein cholesterol (LDL-c) concentrations were measured using an automated analyser (RX Daytona, Randox Laboratories, UK). Reported TAG values in the present paper have been blanked for glycerol based on recommendations for clinical research [39]. Serum insulin and leptin were measured using enzyme-linked immunosorbent assay (ELISA) kits (Mercodia AB, Sweden). Fibroblast growth factor 21 (FGF21) was measured using a U-plex electro-chemiluminescent kit (U-Plex, Mesoscale Discovery, USA). Incremental area under the curve (iAUC) or total area under the curve (tAUC) for postprandial responses were calculated with the trapezoid method using the Time Series Response Analyser [40]. Inter-assay coefficients of variation were < 3% for glucose, < 2% for TAG, < 6% for glycerol, < 7% for NEFA, < 3% for lactate, < 6% for βOHB, < 4% for total cholesterol, < 5% for HDL-c, < 6% for LDL-c, < 7% for insulin, < 6% for leptin, and < 3% for FGF21. All samples for a participant were measured on the same run or plate. Samples producing values below the lower limit of detection were assigned the value of the lower detectable concentration, which was necessary for some samples with βOHB, insulin, and leptin.

### Statistical analyses

Descriptive statistics were calculated using Microsoft Excel (Microsoft, Washington, USA). GraphPad Prism was used for other statistical analyses and producing figures (GraphPad Software Inc., California, USA). The distribution of residuals was checked using Shapiro–Wilk tests and visual inspection of residual plots. Single-variable outcomes were analysed using one-way repeated measures ANOVA with post-hoc Bonferroni corrections applied. Outcomes with multiple time-points for each condition were analysed using two-way repeated measures ANOVA or mixed-effects models (depending on missing data points) to detect significant time, condition, or time x condition interactions, with post hoc Bonferroni corrections applied. Pearson correlation coefficients were used to assess linear associations between βOHB and NEFA, and LDL-cholesterol and NEFA across 4 and 24 h in the LOWCHO condition. The larger sample size than originally planned offered the opportunity to perform post-hoc tests exploring sex differences in physiological outcomes. Firstly, we ran two-way ANOVA to detect significant sex x condition effects for summative outcomes (iAUC and tAUC across 4 or across 24 h for outcomes with only 3 time points) and secondly, we disaggregated data by sex and ran the same analyses as the whole cohort to identify changes in interpretation compared to the whole sample. Significance was accepted at *P* ≤ 0.05. Data are presented as mean and 95% confidence intervals (CI) unless otherwise stated.

## Results

### Physical activity energy expenditure

**Fig. 3:**
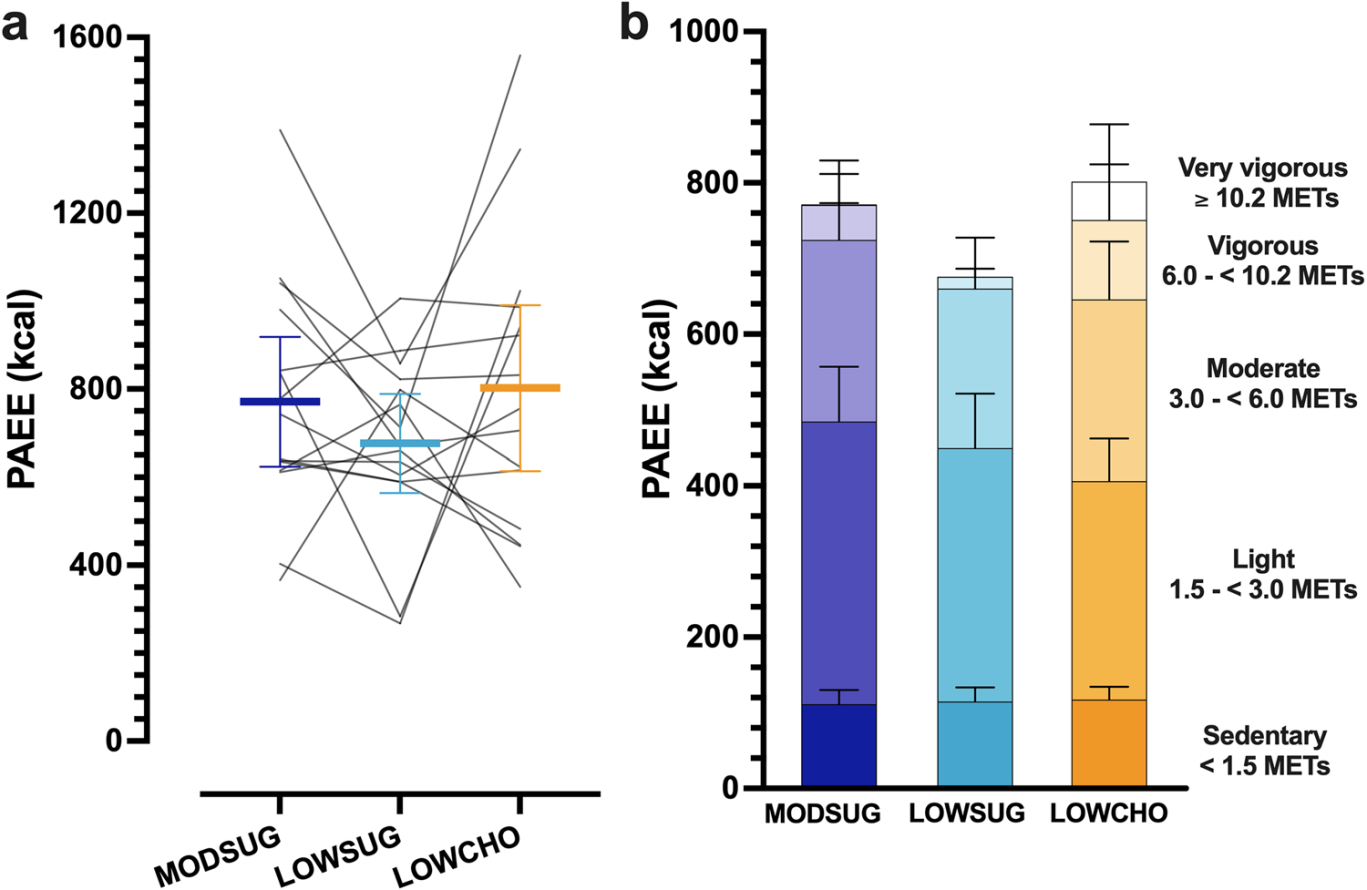
Physical activity energy expenditure (*PAEE*) across 24 h of moderate-sugar diet (*MODSUG*), low-sugar diet (*LOWSUG*), or a low-carbohydrate diet (*LOWCHO*) in healthy men and women. Minute-by-minute PAEE (**a**) and cumulative PAEE split into intensity thresholds (**b**). Habitual PAEE is included from the preliminary week of monitoring to display the impact of the postprandial laboratory testing. *n* = 15. Data expressed as mean ± 95% confidence intervals

Physical activity energy expenditure was not different between conditions (*P* = 0.29; Fig. 3A). When classified into physical activity intensity thresholds, there was an overall condition effect for vigorous intensity physical activity (*P* = 0.03) but following adjustment for multiple comparisons, no differences between conditions were apparent (all *P* > 0.05; Fig. 3B). Pre-trial 24-h physical activity energy expenditure was 1002 (694 to 1309) kcal prior to MODSUG, 870 (700–1040) kcal prior to LOWSUG, and 999 (815–1183) kcal prior to LOWCHO (all *P* > 0.05).

### Energy balance and nutrient intake

**Fig. 4:**
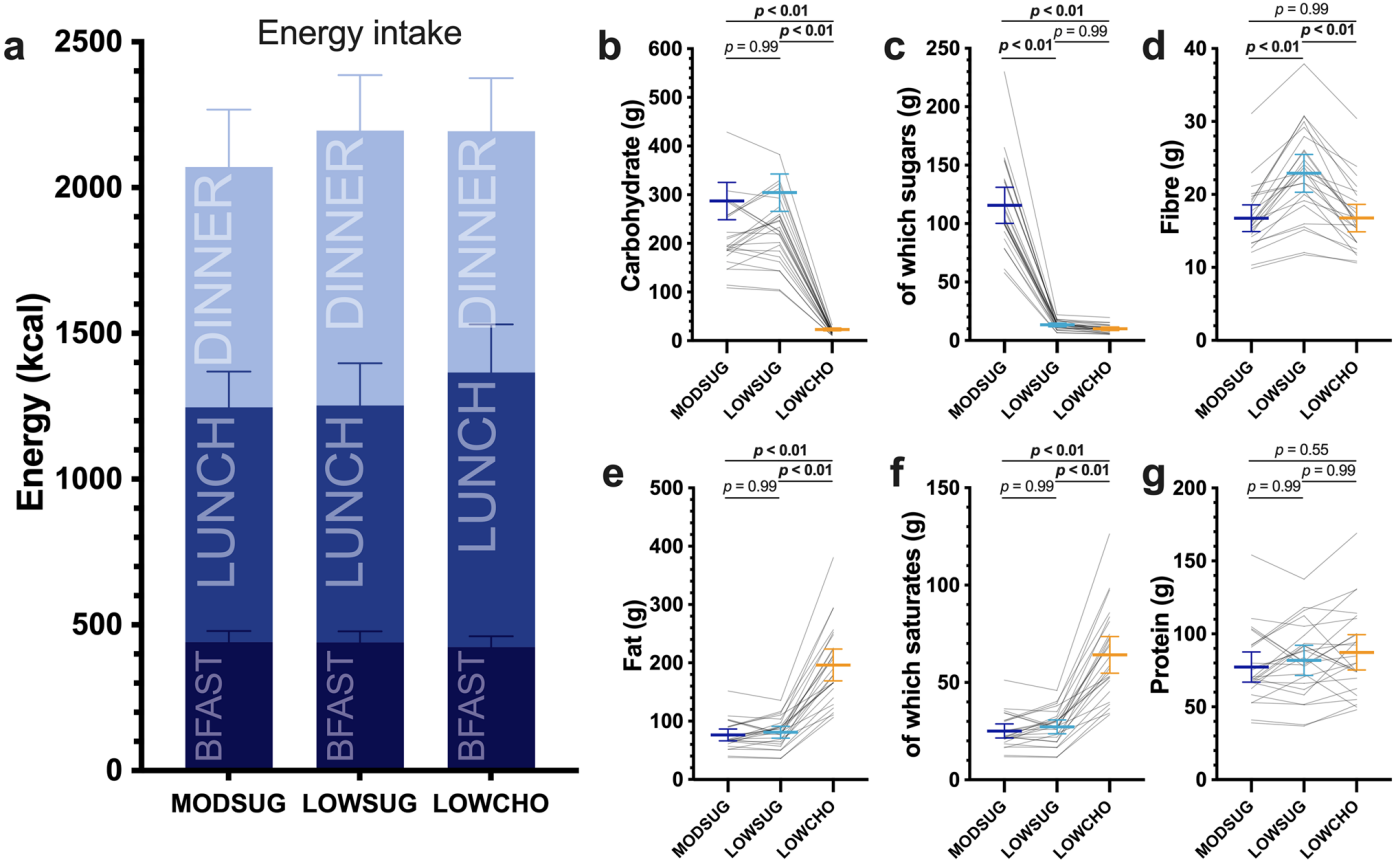
Energy and nutrient intake across 24 h of moderate-sugar diet (*MODSUG*), low-sugar diet (*LOWSUG*), or a low-carbohydrate diet (*LOWCHO*) in healthy men and women. Intakes of energy partitioned by meal (**a**), carbohydrate (**b**), sugars (**c**), fibre (**d**), fat (**e**), saturated fat (**f**), and protein (**g**). *n* = 25. Data expressed as mean ± 95% confidence intervals

Energy intake at lunch, dinner, and across 24 h was similar in all conditions (Fig. 4A). By design, dietary carbohydrate intake was lower in the LOWCHO condition compared to other conditions (Fig. 4B) and dietary fat and saturated fat intake were greater in the LOWCHO condition compared to other conditions (Fig. 4E, F). Dietary sugar intake was greater in the MODSUG condition compared to other conditions (Fig. 4C). Dietary protein intake was well-matched between conditions (Fig. 4G). Dietary fibre intake was 6.2 (2.6–9.8) g higher during LOWSUG vs. MODSUG and 6.1 (2.6–9.7) g higher in LOWSUG vs. LOWCHO (Fig. 4D). Mass of food eaten was 102 (20–185) g greater at dinner and 156 (44–267) g greater across 24 h in the LOWSUG vs. MODSUG (*P* = 0.01 and *P* < 0.01, respectively) and was 111 (23–199) g greater at dinner and 123 (15–231) g greater across 24 h in the LOWSUG vs. LOWCHO (*P* = 0.01 and *P* = 0.02, respectively; Supplemental Fig. 2A). Time taken to eat breakfast was lower during LOWCHO vs. LOWSUG (*P* < 0.01; Supplemental Fig. 2B). Time taken to eat lunch did not differ between conditions (all *P* > 0.05; Supplemental Fig. 2C).

### Substrate oxidation and appetite responses

**Fig. 5:**
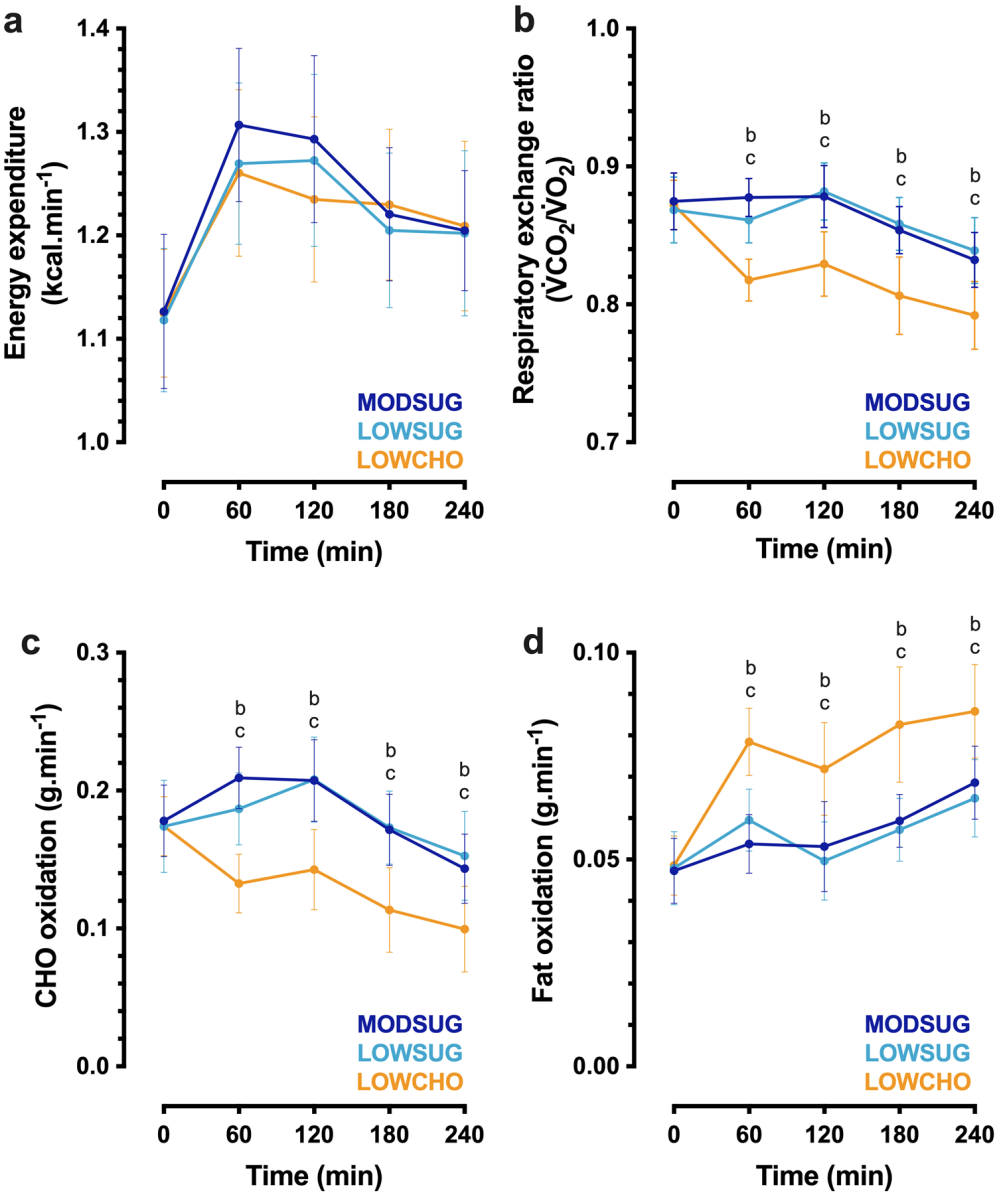
Postprandial energy expenditure (**a**), respiratory exchange ratio (**b**), carbohydrate (*CHO*) oxidation (**c**), and fat oxidation (**d**) responses to a moderate-sugar diet (*MODSUG*), low-sugar diet (*LOWSUG*), or a low-carbohydrate diet (*LOWCHO*) in healthy men and women. *n* = 25. Data expressed as mean ± 95% confidence intervals. Annotations: **b**, *P* < 0.05 MODSUG vs. LOWCHO; **c**, *P* < 0.05 LOWSUG vs. LOWCHO

Following breakfast ingestion, energy expenditure increased (time effect, *P* < 0.001) in all conditions (time x condition interaction, *P* = 0.153; Fig. 5A). Postprandial RER was lower during LOWCHO vs. MODSUG and LOWSUG (time x condition interaction, *P* < 0.001; Fig. 5B). Carbohydrate oxidation rates were lower in the LOWCHO condition vs. MODSUG and LOWSUG (time x condition interaction, *P* < 0.001; Fig. 5C), whereas fat oxidation rates were greater (time x condition interaction, *P* < 0.001; Fig. 5D).

**Fig. 6:**
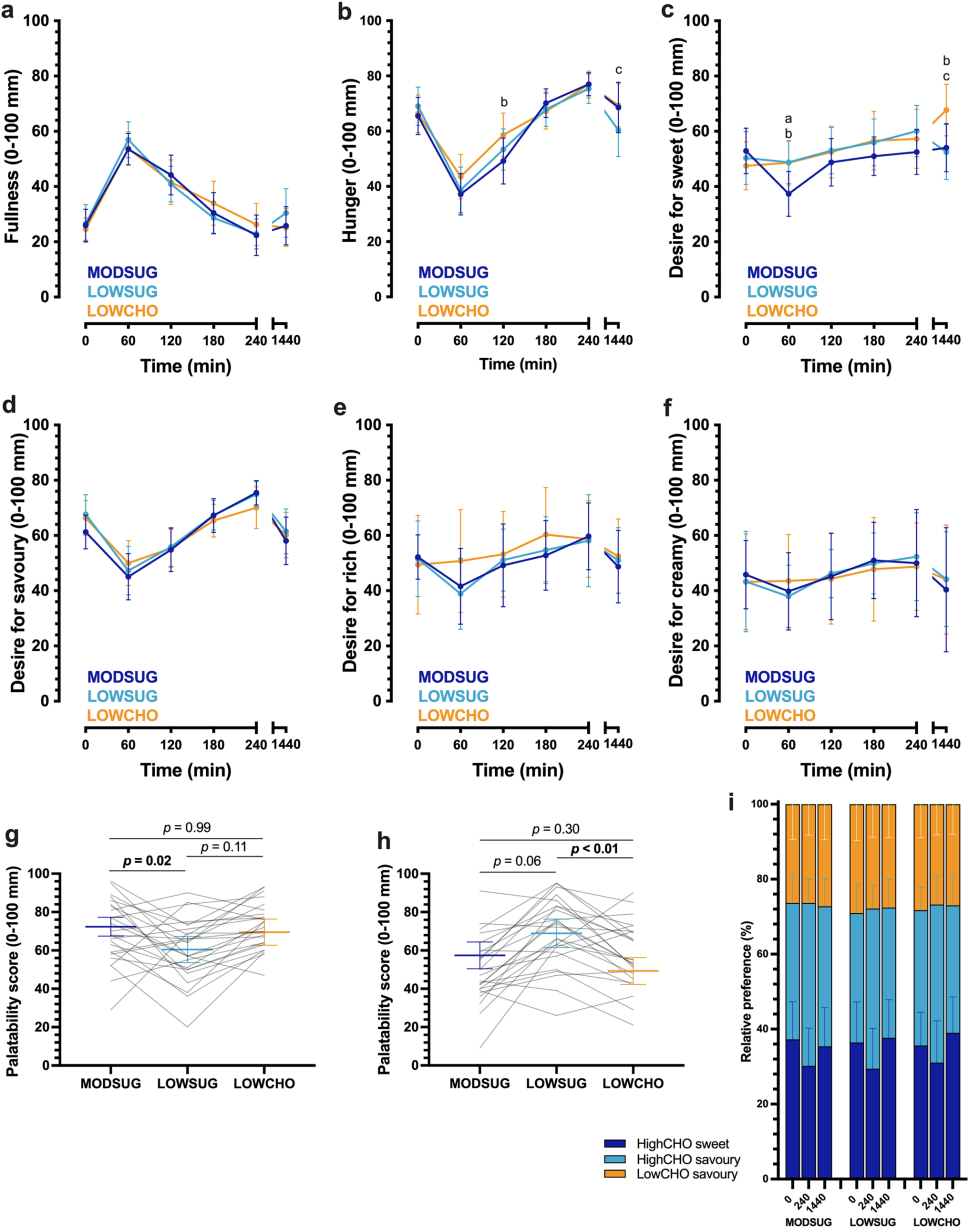
Postprandial and 24-h appetite and palatability responses to a moderate sugar diet (*MODSUG*), low-sugar diet (*LOWSUG*), or a low-carbohydrate diet (*LOWCHO*) in healthy men and women. Postprandial and 24-h visual analogue scale ratings for fullness (**a**), hunger (**b**), desire for sweet (**c**), desire for savoury (**d**), desire for rich (**e**), and desire for creamy (**f**). Palatability visual analogue scale ratings for breakfast (**g**) and lunch (**h**). Relative food preference for high-carbohydrate sweet, high-carbohydrate savoury, or low-carbohydrate savoury foods at baseline, 4 h postprandial, and after 24 h of MODSUG, LOWSUG, and LOWCHO (**i**). *n* = 25. Data expressed as mean ± 95% confidence intervals. Annotations: **a**, *P* < 0.05 MODSUG vs. LOWSUG; **b**, *P* < 0.05 MODSUG vs. LOWCHO; **c**, *P* < 0.05 LOWSUG vs. LOWCHO

Ratings of fullness and hunger were not different between conditions (time x condition interaction, *P* = 0.29; Fig. 6A). Hunger ratings initially declined following breakfast before rising to baseline (time effect, *P* < 0.001), rising more rapidly with MODSUG vs. LOWCHO (time x condition interaction, *P* = 0.034; Fig. 6B). Following 24 h of the diets, hunger ratings were lower with LOWSUG vs. LOWCHO (Fig. 6B). Thirst ratings responded similarly in all conditions (time x condition interaction, *P* = 0.734). There was an initial decrease in desire for sweet foods with MODSUG vs. both LOWSUG and LOWCHO (time x condition interaction, *P* < 0.01; Fig. 6C). In the fasted state following 24 h of the diets, desire for sweet foods was higher with LOWCHO vs. both LOWSUG and MODSUG (Fig. 6C). Ratings of desire for savoury and rich foods initially decreased following breakfast ingestion before rising (time effect, all *P* < 0.001; Fig. 6D, E), whereas desire for creamy foods did not demonstrate such a clear postprandial change (*P* = 0.057; Fig. 6F). Based on the computer task, preference for high carbohydrate savoury food was higher at lunchtime in all conditions (time effect, *P* < 0.001; time x condition interaction, *P* = 0.16; Fig. 6I), and preference for high carbohydrate sweet food was lower at lunchtime in all conditions (time effect, *P* < 0.001; time x condition interaction, *P* = 0.31; Fig. 6I). The MODSUG breakfast was rated as more palatable than LOWSUG (Fig. 6G), and the LOWSUG lunch/dinner was rated as more palatable than LOWCHO (Fig. 6H).

### Systemic metabolic and endocrine responses

**Fig. 7:**
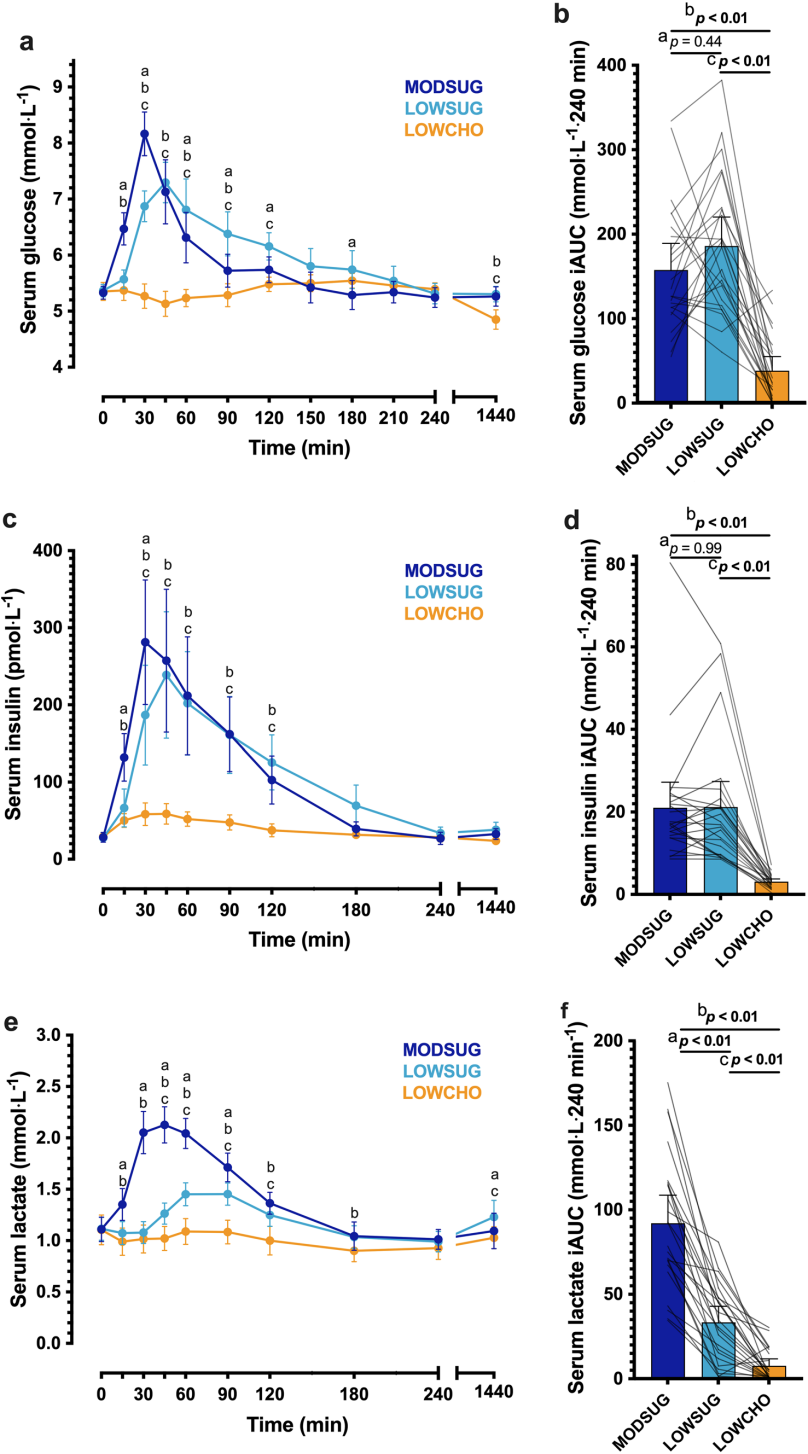
Postprandial and 24-h responses to a moderate sugar diet (*MODSUG*), low-sugar diet (LOWSUG), or a low-carbohydrate diet (*LOWCHO*) in healthy men and women. Time course and incremental area under the curves (*iAUC*) of serum glucose (**a**, **b**), insulin (**c**, **d**), and lactate (**e**, **f**). *n* = 24. Data expressed as mean ± 95% confidence intervals. Annotations: **a**, *P* < 0.05 MODSUG vs. LOWSUG; **b**, *P* < 0.05 MODSUG vs. LOWCHO; **c**, *P* < 0.05 LOWSUG vs. LOWCHO

Following breakfast ingestion, serum glucose, insulin and lactate concentrations rose to a greater extent with MODSUG vs. LOWSUG (time x condition interaction, both *P* < 0.001; Fig. 7A, C, E). In contrast, serum glucose, insulin and lactate concentrations remained essentially at fasting concentrations during LOWCHO (Fig. 7A, C, E). This resulted in higher peak serum glucose, insulin, and lactate concentrations with MODSUG vs. LOWSUG and higher with LOWSUG vs. LOWCHO, respectively (Table 4). Postprandial serum glucose and insulin iAUC were higher with MODSUG and LOWSUG vs. LOWCHO with no differences between MODSUG and LOWSUG (Fig. 7B, D). In contrast, postprandial serum lactate iAUC was higher with MODSUG vs. LOWSUG, and higher with LOWSUG vs. LOWCHO (Fig. 7F). In the fasted state 24 h following initiation of the diets, serum glucose concentrations were lower with LOWCHO vs. both MODSUG and LOWSUG (Fig. 7A).

**Table 4:** Peak glucose, insulin, and lactate concentrations in the 4-h postprandial to MODSUG, LOWSUG or LOWCHO breakfasts

	MODSUG	LOWSUG	LOWCHO	
Peak serum glucose (mmol L^−1^)	8.33 (7.98–8.68)	7.78 (7.46–8.09)	5.69 (5.58–5.80)	MODSUG vs. LOWSUG *P* = 0.01 MODSUG vs. LOWCHO *P* < 0.001 LOWSUG vs. LOWCHO *P* < 0.001
Peak serum insulin (pmol L^−1^)	322 (231–413)	292 (206–379)	72 (58–85)	MODSUG vs. LOWSUG *P* = 0.28 MODSUG vs. LOWCHO *P* < 0.001 LOWSUG vs. LOWCHO *P* < 0.001
Peak serum lactate (mmol L^−1^)	2.26 (2.08–2.44)	1.54 (1.44–1.65)	1.28 (1.15–1.41)	MODSUG vs. LOWSUG *P* < 0.001 MODSUG vs. LOWCHO *P* < 0.001 LOWSUG vs. LOWCHO *P* < 0.001

Data are mean (95% confidence intervals)

**Fig. 8:**
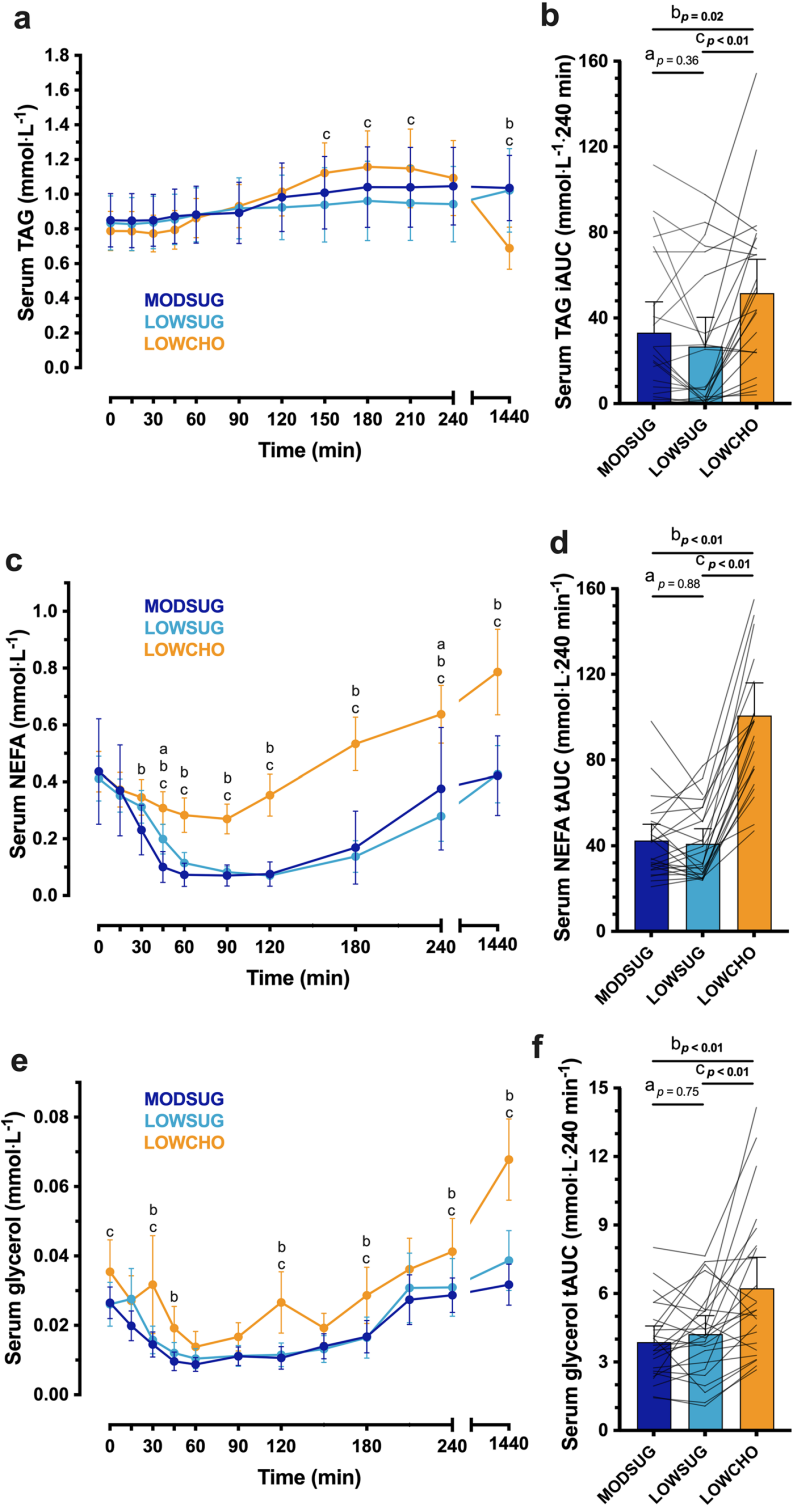

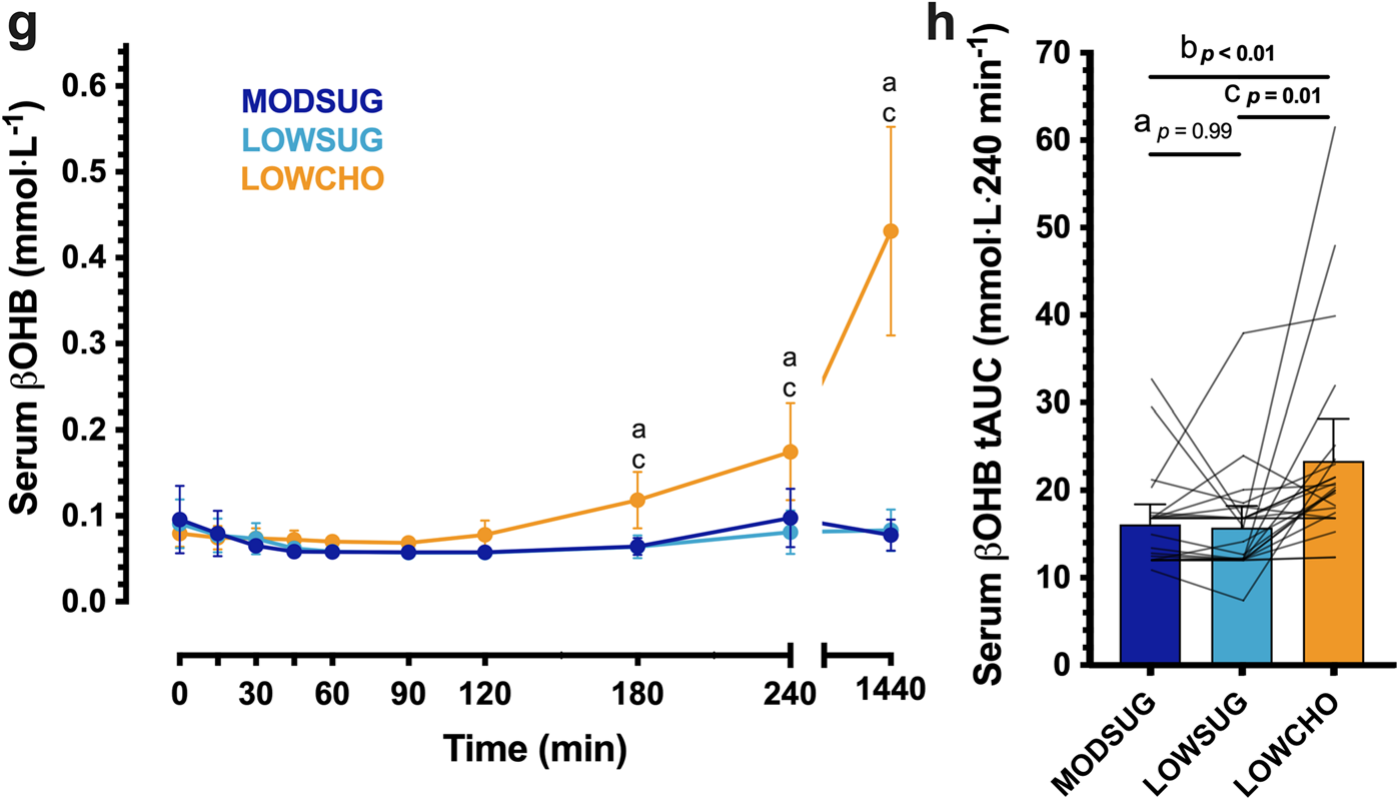
Postprandial and 24-h responses to a moderate-sugar diet (*MODSUG*), low-sugar diet (LOWSUG), or a low-carbohydrate diet (*LOWCHO*) in healthy men and women. Time course and incremental area under the curves (*iAUC*) of serum triglycerides (**a**, **b**), non-esterified fatty acids (**c**, **d**), glycerol (**e**, **f**), and β-hydroxybutyrate (**g**, **h**). *n* = 24. Data expressed as mean ± 95% confidence intervals. Annotations: **a**, *P* < 0.05 MODSUG vs. LOWSUG; **b**, *P* < 0.05 MODSUG vs. LOWCHO; **c**, *P* < 0.05 LOWSUG vs. LOWCHO

Following breakfast ingestion, serum TAG concentrations rose to a greater extent with LOWCHO vs. LOWSUG (time x condition interaction, *P* < 0.001; Fig. 8A), leading to a serum TAG iAUC which was greater with LOWCHO vs. both MODSUG and LOWSUG (*P* = 0.02 and *P* < 0.01, respectively; Fig. 8B). In the fasted state 24 h following initiation of the diets, serum TAG concentrations were lower with LOWCHO vs. both MODSUG and LOWSUG (Fig. 8A). Following breakfast ingestion, serum NEFA and glycerol concentrations both decreased to a greater extent with MODSUG and LOWSUG vs. LOWCHO (time x condition interaction, both *P* < 0.001; Fig. 8C, E), leading to serum NEFA and glycerol tAUCs which were greater with LOWCHO vs. both MODSUG and LOWSUG (all *P* < 0.001; Fig. 8D, F). Serum βOHB concentrations began to increase from 180 min following breakfast ingestion (time effect, *P* < 0.001) only in the LOWCHO condition (time x condition interaction, *P* < 0.001; Fig. 8G). This resulted in a postprandial βOHB tAUC which was greater with LOWCHO vs. both MODSUG and LOWSUG (both *P* < 0.001; Fig. 8H). NEFA tAUC was positively correlated with βOHB tAUC across 4- and 24-h (Supplemental Fig. 4A, C) but was not correlated with 4-h and 24-h LDL-cholesterol tAUC (Supplemental Fig. 4B, D).

**Fig. 9:**
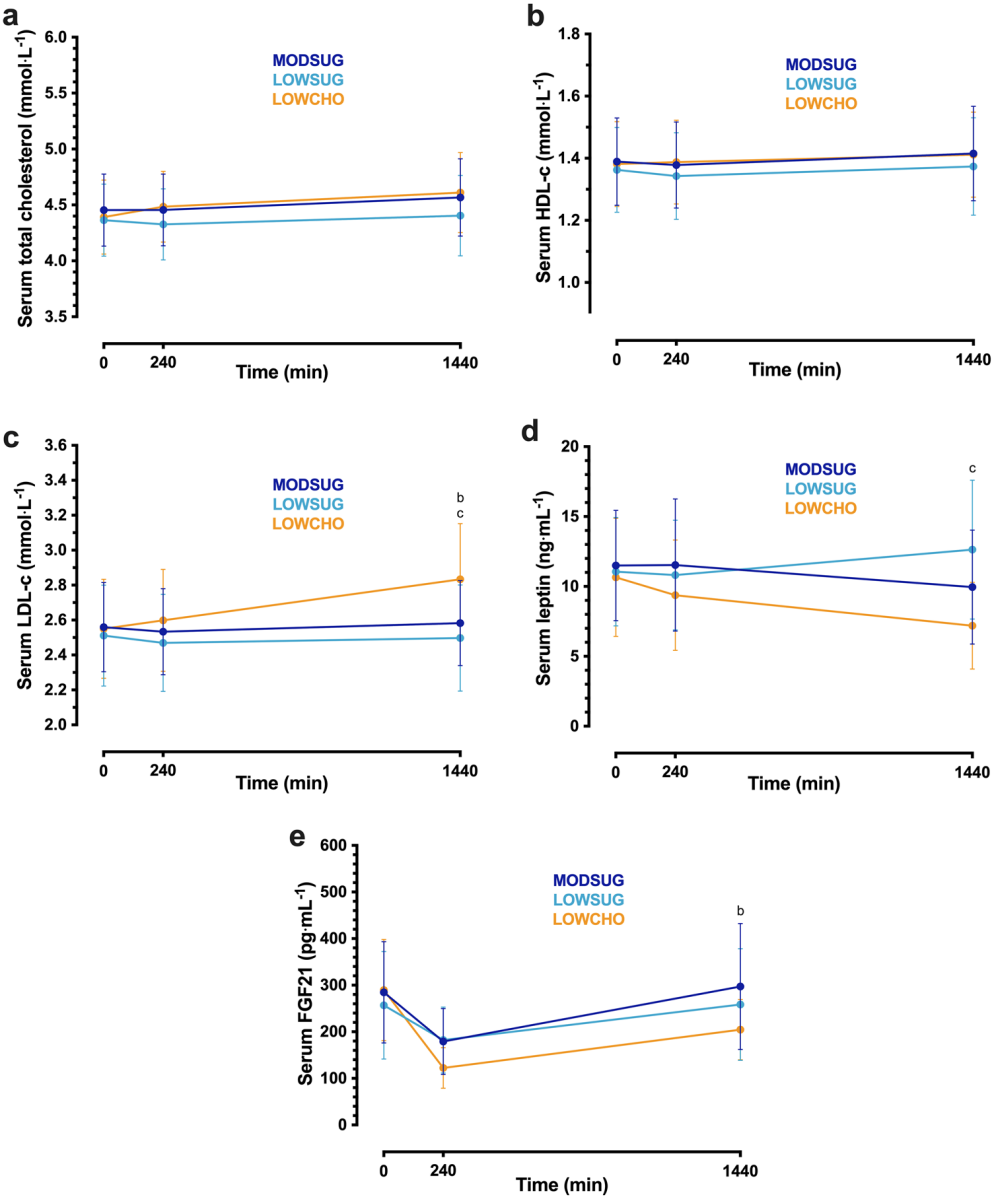
Serum total cholesterol (**a**), HDL-cholesterol (**b**), LDL-cholesterol (**c**), leptin (**d**) and FGF21 (**e**) responses to a moderate sugar diet (*MODSUG*), low-sugar diet (*LOWSUG*), or a low-carbohydrate diet (*LOWCHO*) in healthy men and women. *n* = 24. Data expressed as mean ± 95% confidence intervals. Annotations: **b**, *P* < 0.05 MODSUG vs. LOWCHO; **c**, *P* < 0.05 LOWSUG vs. LOWCHO

Serum total cholesterol, HDL-cholesterol and LDL cholesterol concentrations all rose across the 24 h period (time effect, *P* = 0.002, *P* = 0.021, and *P* < 0.001, respectively; Fig. 9A–C). The increase in LDL-cholesterol concentrations was greater with LOWCHO vs. both MODSUG and LOWSUG (time x condition interaction, *P* < 0.001; Fig. 9C). Serum leptin concentrations displayed divergent responses between conditions such that by the end of the 24 h period, leptin concentrations were lower with LOWCHO vs. LOWSUG (time x condition interaction, *P* < 0.01; Fig. 9D). Following breakfast ingestion, serum FGF21 concentrations decreased in all conditions and remained lower following 24 h of LOWCHO vs. MODSUG (time x condition interaction, *P* < 0.001; Fig. 9E).

### Sex differences

**Fig. 10:**
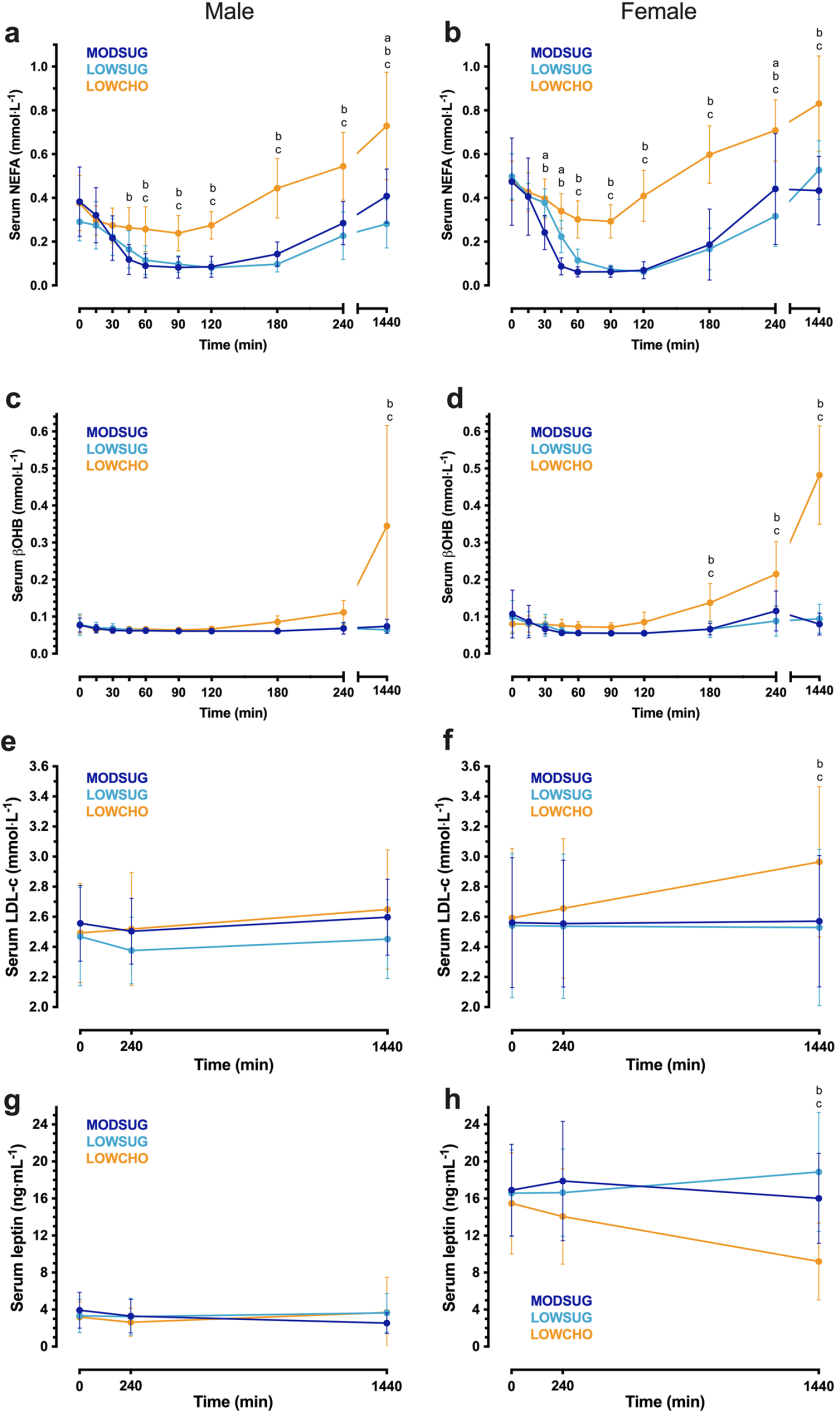
Sex-disaggregated postprandial and 24-h responses to a moderate-sugar diet (*MODSUG*), low-sugar diet (*LOWSUG*), or a low-carbohydrate diet (*LOWCHO*) in healthy men and women. Time course of serum non-esterified fatty acids (**a**, **b**)**,** β-hydroxybutyrate (**c**, **d**), LDL-cholesterol (**e**, **f**), and leptin (**g**, **h**). Females *n* = 14, males *n* = 10. Data expressed as mean ± 95% confidence intervals. Annotations: **a**, *P* < 0.05 MODSUG vs. LOWSUG; **b**, *P* < 0.05 MODSUG vs. LOWCHO; **c**, *P* < 0.05 LOWSUG vs. LOWCHO

There was a trend towards higher NEFA tAUC (sex effect, *P* = 0.06, sex x condition interaction, *P* = 0.08) and βOHB tAUC (sex effect, *P* = 0.06, sex x condition interaction, *P* = 0.10) in females compared to males, but analysis of responses within each sex did not change interpretation of the findings (Fig. 10A–D). Females did not display higher LDL-cholesterol iAUC vs. males (sex effect, *P* = 0.11, sex x condition interaction, *P* = 0.28), but analysis of responses within each sex revealed the increase in LDL-cholesterol across 24-h in LOWCHO was driven primarily by females (male time x condition interaction, *P* = 0.12, female time x condition interaction, *P* < 0.001, Fig. 10E, F). Serum leptin tAUC was higher in females than males (sex effect, *P* < 0.001, sex x condition interaction, *P* = 0.008) and analysis of responses within each sex revealed the decrease in leptin across 24-h in LOWCHO was driven primarily by females (male time x condition interaction, *P* = 0.33, female time x condition interaction, *P* < 0.001, Fig. 10G, H). Serum TAG iAUC was lower in females than males (sex effect, *P* < 0.001, sex x condition interaction, *P* = 0.86), but analysis of responses within each sex did not change interpretation of the findings (Supplemental Fig. 3A, B). Serum lactate iAUC following MODSUG was lower in females than males (sex x condition interaction, *P* = 0.04; Supplemental Fig. 3C, D) and serum glycerol was greater in females than males (sex effect, *P* = 0.005, sex x condition interaction, *P* = 0.85). Analysis of responses within each sex revealed the decrease in FGF21 across 24-h in LOWCHO was driven primarily by females (male time x condition interaction, *P* = 0.14, female time x condition interaction, *P* = 0.008, Supplemental Fig. 3E, F). There were no main effects of sex or sex x condition interactions for other outcomes (all P > 0.05, Supplemental Fig. 3) and the interpretation of other physiological outcomes did not change when analysing data within each sex, but some time-points were changed.

## Discussion

The present study reveals that total physical activity energy expenditure is not detectably altered by either restricting free sugars to less than 5% of energy intake or restricting overall carbohydrate intake to less than 8% of energy intake in the initial 24 h (~ 19 h free-living), despite distinct physiological responses with ketogenic carbohydrate restriction. Manipulating the type and/or amount of dietary carbohydrate also did not alter energy intake across 24 h. Twenty-four hours of a low-carbohydrate ketogenic diet did, however, reduce serum glucose, insulin, triglyceride, leptin, and FGF21 concentrations, and increased βOHB, NEFA, and LDL-cholesterol concentrations.

In the present study, no detectable effect of carbohydrate or sugar restriction on directly measured physical activity energy expenditure was observed in the initial 24-h of commencing the diets (of which ~ 19 h were free-living). Prior work has indicated that carbohydrate availability may alter physical activity as demonstrated by energy expenditure in response to genetically increased hepatic glycogen stores in mice [18], and lower physical activity with alternate day fasting and breakfast skipping in humans [8, 17]. Indeed, a reduction in the ratio of total energy expenditure to resting metabolic rate has been observed when people consumed a low-carbohydrate ketogenic diet versus a high-carbohydrate diet [20], indicating a potential effect on physical activity. The lack of detectable changes in directly measured physical activity with acute carbohydrate or sugar restriction in the present study indicate that the acute responses seen with intermittent fasting and breakfast skipping may be primarily driven by energy intake rather than carbohydrate intake. The reduction in indirectly measured physical activity with carbohydrate restriction that has previously been observed [20], suggests that either the potential effects of carbohydrate restriction on physical activity occur in the longer-term, and(or) that components of energy expenditure other than physical activity and resting metabolic rate are decreased with carbohydrate restriction. There is debate around the propensity for carbohydrate restriction to alter total energy expenditure, with suggestions that duration might be an important factor [41, 42]. Findings from the present study suggest physical activity is not a large contributor to potential changes in total energy expenditure in the initial 24 h, which agrees with findings from 5 days of carbohydrate-manipulated diet in overweight men [43]—although not to ketogenic levels. Furthermore, it is possible that a decrease in physical activity erodes the potential energy deficit arising from sugar restriction, consequent from adhering to government guidelines, but the present study suggests this does not occur across 24 h, providing initial support for the guidelines regarding physical activity.

The absence of any meaningful differences in physical activity energy expenditure was observed in the presence of similar 24-h energy intake between conditions. In a manner designed to enhance external validity, ad libitum intake of lunch and dinner was permitted and, despite differences in nutrient composition and palatability, 24-h energy intake did not differ between conditions. Energy density was relatively closely matched between conditions. Room calorimetry studies have shown that large differences in energy density (from 1.15 to 1.68 kcal g^−1^) with high-fat, low-carbohydrate versus high-carbohydrate, low-fat diets increase energy intake linearly (from 2158 kcal d^−1^ to 2954 kcal d^−1^) [44]; however, when energy density is matched, dietary macronutrient manipulation typically does not change energy intake [45]. Similarly, it has been proposed that diets high in sugar lead to increases in body mass via increased energy intake, which is thought to be explained, at least in part, by increasing dietary energy density [10]. Therefore, the lack of difference in energy intake in the present study is most likely due to the matching of energy density.

The similar energy intake and physical activity between conditions in the present study were observed in the presence of substantial changes in substrate metabolism and hormone availability linked to energy balance behaviours. Considerable decreases in FGF21 (85 pg mL^−1^) and leptin (3.5 ng mL^−1^) concentrations were observed with 24 h of carbohydrate (but not sugar) restriction, alongside markedly increased βOHB (0.35 mmol L^−1^). Diet-induced ketogenesis achieving βOHB concentrations ~ 0.5 mmol L^−1^ after 4–8 weeks are associated with increased subjective feelings of satiety and fullness and decreased hunger and desire to eat [46]; however, similar concentrations of βOHB in the present study did not translate into changes in appetite ratings after 24 h. FGF21 concentrations increase postprandially in humans with infusion or ingestion of sugars [47, 48]. Interestingly, however, carbohydrate restriction and not sugar restriction per se lowered 24-h FGF21 and glucose concentrations in the present study, and this decrease in FGF21 concentrations in the LOWCHO condition coincided with reduced fasting glucose and an increase in desire for sweet food after 24 h, consistent with evidence in mice that FGF21 administration inhibits sucrose intake via paraventricular nerve signalling [47]. Leptin is an adipokine involved in the long-term regulation of energy balance and adipose tissue mass [49]. Three days of carbohydrate restriction (from 70 to 35% of energy intake) can decrease leptin concentrations by ~ 1.5 ng mL^−1^ in postmenopausal women [50], and 4-weeks of ketogenic diet reduce serum leptin concentrations by ~ 1.8 ng mL^−1^ in men with overweight or obesity compared to an isoenergetic diet containing ~ 50% carbohydrate [51]. We observed substantial decreases in leptin concentrations of ~ 3.5 ng mL^−1^ after just 24 h of carbohydrate restriction, which is likely due to reduced glycaemic and(or) insulinaemic responses [52]. The lack of changes in energy intake suggests that the changes in ketone body, FGF21 and leptin availability, are insufficient to alter energy intake with carbohydrate restriction in the first 24-h of following such diets.

In the present study, carbohydrate (but not sugar) restriction lowered postprandial glucose and insulin concentrations alongside carbohydrate oxidation rates, whilst raising postprandial TAG, NEFA and glycerol concentrations alongside greater fat oxidation rates. The following morning, fasting serum TAG concentrations were markedly reduced with carbohydrate restriction. The higher immediate postprandial TAG concentrations and the lower fasting TAG concentrations the following morning are likely explained by the increased fat intake (thus greater chylomicron appearance), as well as less suppression of VLDL-TAG due to lower insulinaemia, as insulin suppresses VLDL secretion directly and indirectly [53, 54]. The lower fasting TAG concentrations the next morning likely reflects increased TAG uptake by muscle when carbohydrate availability is low. Whilst this response has been shown following 3 days of reduced-carbohydrate diet [55], here we demonstrate this response can occur within 24 h with severe carbohydrate restriction.

Serum lactate was the only metabolic effect measured, which responded specifically to sugar restriction, whereby postprandial lactate concentrations were lowered by sugar restriction, and lowered further still by total carbohydrate restriction. Whereas ingestion of any carbohydrates in sufficient amounts will stimulate carbohydrate utilization and thus can increase lactate concentrations, the higher lactate concentrations with sugar ingestion are likely to reflect hepatic fructose metabolism [56]. High fructose intake results in rapid and unregulated flux of fructose through the liver, leading to accumulation of triose phosphate, which can be converted into triglycerides, lactate and/or glucose [56]. Therefore the higher lactate concentrations observed with higher sugar intake likely reflect hepatic accumulation of triose phosphate [56], and therefore (under sedentary conditions) might be expected to stimulate de novo lipogenesis and serum triglyceride concentrations. However, while very high fructose ingestion can stimulate de novo lipogenesis and increase plasma triglyceride concentrations [57], our data demonstrate that—at least in healthy men and women, over a short time frame—typical intakes of sugar do not raise serum triglyceride concentrations when compared to very low sugar intakes.

To the best of the authors knowledge, this is the first evidence that a low-carbohydrate ketogenic diet can raise LDL-cholesterol concentrations over such a short timeframe (24 h), despite similar total energy intake between conditions. The reduced insulin concentrations and increased NEFA concentrations observed in the present study are likely to have increased VLDL production [58], and LDL-cholesterol is produced by the hydrolysis of VLDL as VLDL particles become lipid depleted [59]. Therefore, higher VLDL concentrations from increased fatty acid availability across the day is a likely explanation for the increase in LDL-cholesterol. Interestingly, the increase in LDL-cholesterol across 24 h occurred primarily in female participants. The trend towards higher NEFA concentrations in females may have provided more precursor for VLDL production [58]. However, it has also been shown that despite similar postprandial VLDL concentrations between males and females [60], females partition NEFA to βOHB (rather than to VLDL) to a greater extent than males [61]. Our data support this, as NEFA responses were positively correlated with βOHB responses across 4 and 24 h, but not with LDL-cholesterol. Furthermore, females have increased adipose tissue postprandial lipoprotein lipase activity versus males [62]. Altogether, this suggests the increased LDL-cholesterol concentrations driven by females are explained by increased LPL-mediated hydrolysis of VLDL rather than increased VLDL production.

In addition to sex-specific responses with LDL cholesterol, our data demonstrate that carbohydrate restriction causes a reduction in leptin concentrations in females but not in males. Leptin concentrations were considerably lower in male participants than females in the present study, which is consistent with previous literature and appears to be only partly explained by body fat mass [63, 64]. This was an exploratory discovery which provokes consideration of sex when investigating hormonal responses to dietary carbohydrate manipulation.

Whilst a strength of the study is providing real foods in their natural matrix, rather than meal replacements or supplements, it should be noted that the results presented are specific to the context of the foods provided, and not necessarily representative of different foods which fit the same macronutrient profile. This highlights the requirement for multiple studies to validate findings when investigating the effects of macronutrient manipulation per se. Furthermore, the laboratory confinement following the breakfast meal may have impacted the interpretation of 24-h physical activity energy expenditure by influencing a period where physical activity could have varied. Whilst this was deemed necessary for the present study, to provide context about the physiological stimuli of each diet, future studies should include measurement of physical activity in the immediate postprandial period following breakfast.

In summary, these data demonstrate that, when energy density is controlled for, the amount of dietary carbohydrate has little impact on physical activity and energy intake in the initial 24 h (~ 19 h free-living). This is despite rapid, profound changes in circulating metabolites and appetite hormones with total carbohydrate restriction. These data also demonstrate that restricting sugar intake from 20 to 5% of energy intake does not acutely alter postprandial triglyceride or LDL-cholesterol concentrations nor fasting triglyceride or LDL-cholesterol concentrations the following morning. Conversely, carbohydrate restriction lowered triglyceride concentrations after 24 h but markedly increased LDL-cholesterol concentrations. Interestingly, the largest changes in leptin and LDL-cholesterol concentrations occurred in female participants. Together, these data suggest that specifically reducing dietary sugar intake does not acutely alter energy intake or metabolic health markers, at least within the context of a healthy body mass. Since many of the hormonal responses were more clearly observed after 24 h of dietary manipulation, the longer-term effects of these diets require further examination.

## Supplementary Information

Below is the link to the electronic supplementary material.

Supplementary file1 (DOC 219 KB)

Supplementary file2 (DOC 29 KB)

Supplementary file3 (PDF 2828 KB)

Supplementary file4 (PDF 229 KB)

Supplementary file5 (PDF 1269 KB)

Supplementary file6 (PDF 200 KB)
