# Supplementary material for: Restricting sugar or carbohydrate intake does not impact physical activity level or energy intake over 24 h despite changes in substrate use: a randomised crossover study in healthy men and women
Source: Eur J Nutr. 2022 Nov 3;62(2):921–40. doi: 10.1007/s00394-022-03048-x (PMC9941259; doi:10.1007/s00394-022-03048-x)
Supplement: Supplementary file 2 — Supplementary file2 (DOC 29 KB) [file 394_2022_3048_MOESM2_ESM.doc]

**CONSORT 2010 Flow Diagram**

**Allocation**

**Analysis**

**Follow-Up**

**Enrollment**

Assessed for eligibility (n= 33)

Excluded (n= 0)

Lost to follow-up (n= 5):

♦ COVID-19 restrictions (n= 3)

♦ Work schedule too busy (n= 2)

Allocated to intervention (n= 33)

♦ Received allocated intervention (n= 30)

♦ Did not receive allocated intervention:

Stopped replying to correspondence (n= 3)

Analysed (n= 25)

Randomized (n= 33)
