# Supplementary material for: Restricting sugar or carbohydrate intake does not impact physical activity level or energy intake over 24 h despite changes in substrate use: a randomised crossover study in healthy men and women
Source: Eur J Nutr. 2022 Nov 3;62(2):921–40. doi: 10.1007/s00394-022-03048-x (PMC9941259; doi:10.1007/s00394-022-03048-x)
Supplement: Supplementary file 3 — Supplementary file3 (PDF 2828 KB) [file 394_2022_3048_MOESM3_ESM.pdf]

Restricting sugar or carbohydrate intake does not impact physical activity level or energy intake over 24 hours despite changes in substrate use: a randomised crossover study in healthy men and women – Hengist et al. *Eur J Nutr* – Corresponding Author: Javier T. Gonzalez, University of Bath; J.T.Gonzalez@bath.ac.uk

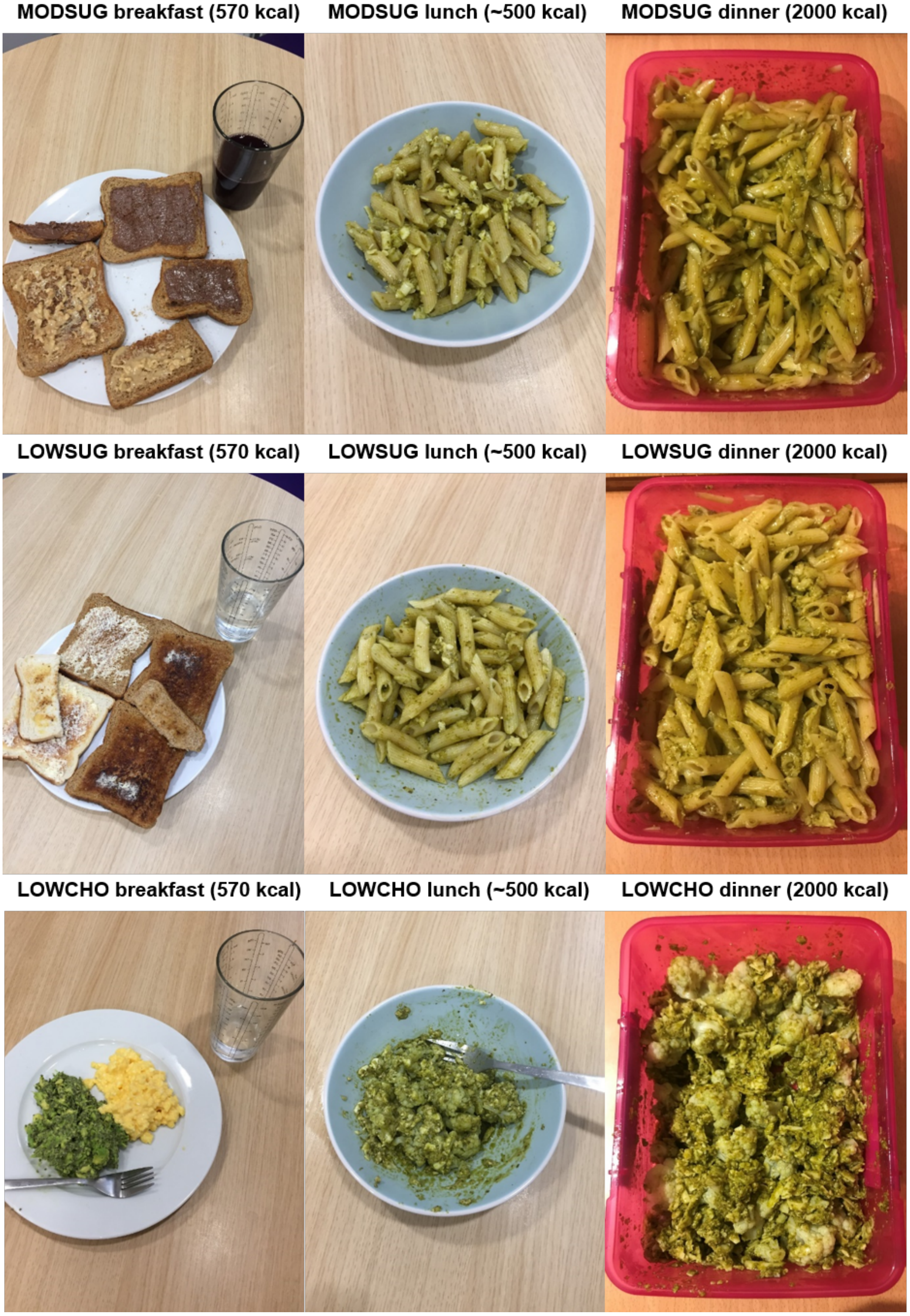

Supplemental Figure 1. Visual demonstrations of breakfast, lunch, and dinner meals provided as part of the moderate sugar (MODSUG), low sugar (LOWSUG), or low carbohydrate (LOWCHO) diets. A representative breakfast of 570 kcal is presented for all diets but the energy content varied for each participant. The lunch meals were partitioned into ~500 kcal portions with deliberate variance, as was presented to participants on trial days. Dinner meals containing ~2000 kcal were provided to participants (the nutritional values of meals were not disclosed to participants).
