# Supplementary material for: Restricting sugar or carbohydrate intake does not impact physical activity level or energy intake over 24 h despite changes in substrate use: a randomised crossover study in healthy men and women
Source: Eur J Nutr. 2022 Nov 3;62(2):921–40. doi: 10.1007/s00394-022-03048-x (PMC9941259; doi:10.1007/s00394-022-03048-x)
Supplement: Supplementary file 4 — Supplementary file4 (PDF 229 KB) [file 394_2022_3048_MOESM4_ESM.pdf]

Restricting sugar or carbohydrate intake does not impact physical activity level or energy intake over 24 hours despite changes in substrate use: a randomised crossover study in healthy men and women – Hengist et al. *Eur J Nutr* – Corresponding Author: Javier T. Gonzalez, University of Bath; J.T.Gonzalez@bath.ac.uk

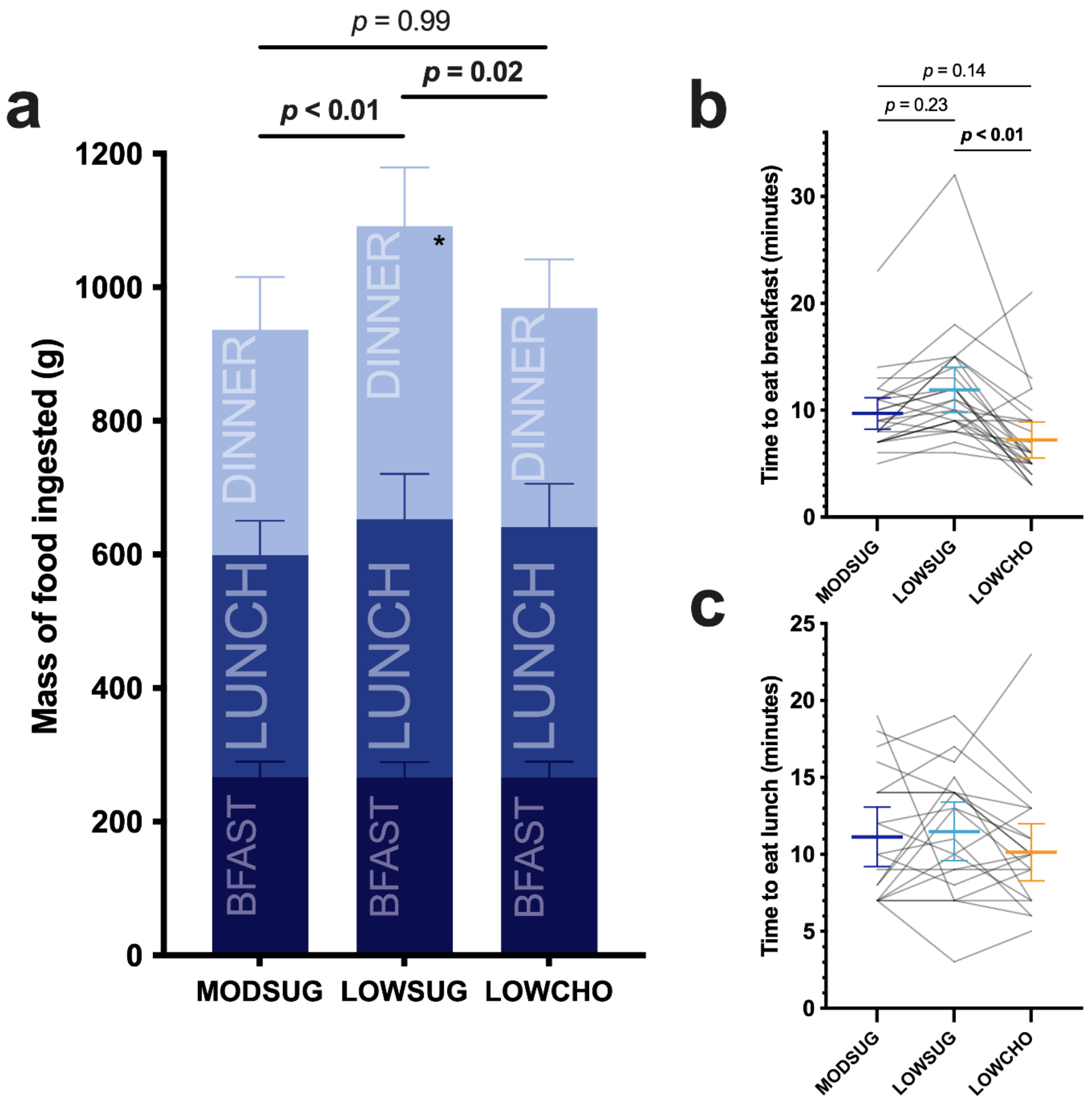

Supplemental Figure 2. Food mass eaten and time taken to consume meals during a moderate sugar diet (MODSUG), low sugar diet (LOWSUG), or a low carbohydrate diet (LOWCHO) in healthy men and women. Mass of food partitioned by meal (a), and time taken to eat breakfast (b) and lunch (c). n = 25. Data expressed as mean ± 95% confidence intervals. *$P < 0.05$ LOWSUG dinner *vs* other conditions.
