# Supplementary material for: Restricting sugar or carbohydrate intake does not impact physical activity level or energy intake over 24 h despite changes in substrate use: a randomised crossover study in healthy men and women
Source: Eur J Nutr. 2022 Nov 3;62(2):921–40. doi: 10.1007/s00394-022-03048-x (PMC9941259; doi:10.1007/s00394-022-03048-x)
Supplement: Supplementary file 5 — Supplementary file5 (PDF 1269 KB) [file 394_2022_3048_MOESM5_ESM.pdf]

Restricting sugar or carbohydrate intake does not impact physical activity level or energy intake over 24 hours despite changes in substrate use: a randomised crossover study in healthy men and women – Hengist et al. *Eur J Nutr* – Corresponding Author: Javier T. Gonzalez, University of Bath; J.T.Gonzalez@bath.ac.uk

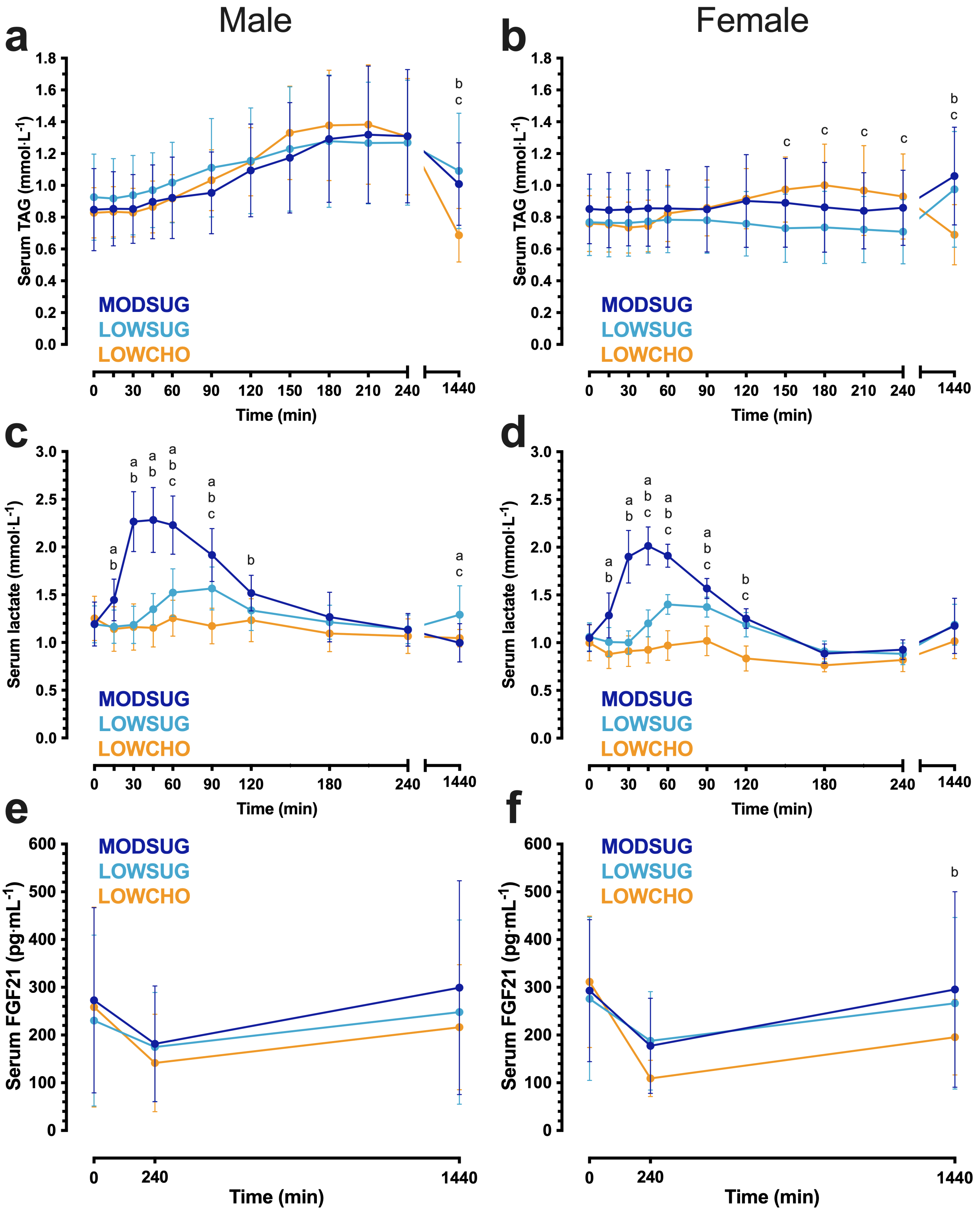

Supplemental Figure 3. Sex-disaggregated postprandial and 24-h responses to a moderate sugar diet (MODSUG), low sugar diet (LOWSUG), or a low carbohydrate diet (LOWCHO) in healthy men and women. Time course of serum triglycerides (a, b), lactate (c, d), and FGF21 (e, f). Females n = 14, males n = 10. Data expressed as mean ± 95% confidence intervals. Annotations: a, $P < 0.05$ MODSUG *vs* LOWSUG; b, $P < 0.05$ MODSUG *vs* LOWCHO; c, $P < 0.05$ LOWSUG *vs* LOWCHO.
