# Supplementary material for: Restricting sugar or carbohydrate intake does not impact physical activity level or energy intake over 24 h despite changes in substrate use: a randomised crossover study in healthy men and women
Source: Eur J Nutr. 2022 Nov 3;62(2):921–40. doi: 10.1007/s00394-022-03048-x (PMC9941259; doi:10.1007/s00394-022-03048-x)
Supplement: Supplementary file 6 — Supplementary file6 (PDF 200 KB) [file 394_2022_3048_MOESM6_ESM.pdf]

**Restricting sugar or carbohydrate intake does not impact physical activity level or energy intake over 24 hours despite changes in substrate use: a randomised crossover study in healthy men and women –** Hengist et al. *Eur J Nutr* – Corresponding Author: Javier T. Gonzalez, University of Bath; J.T.Gonzalez@bath.ac.uk

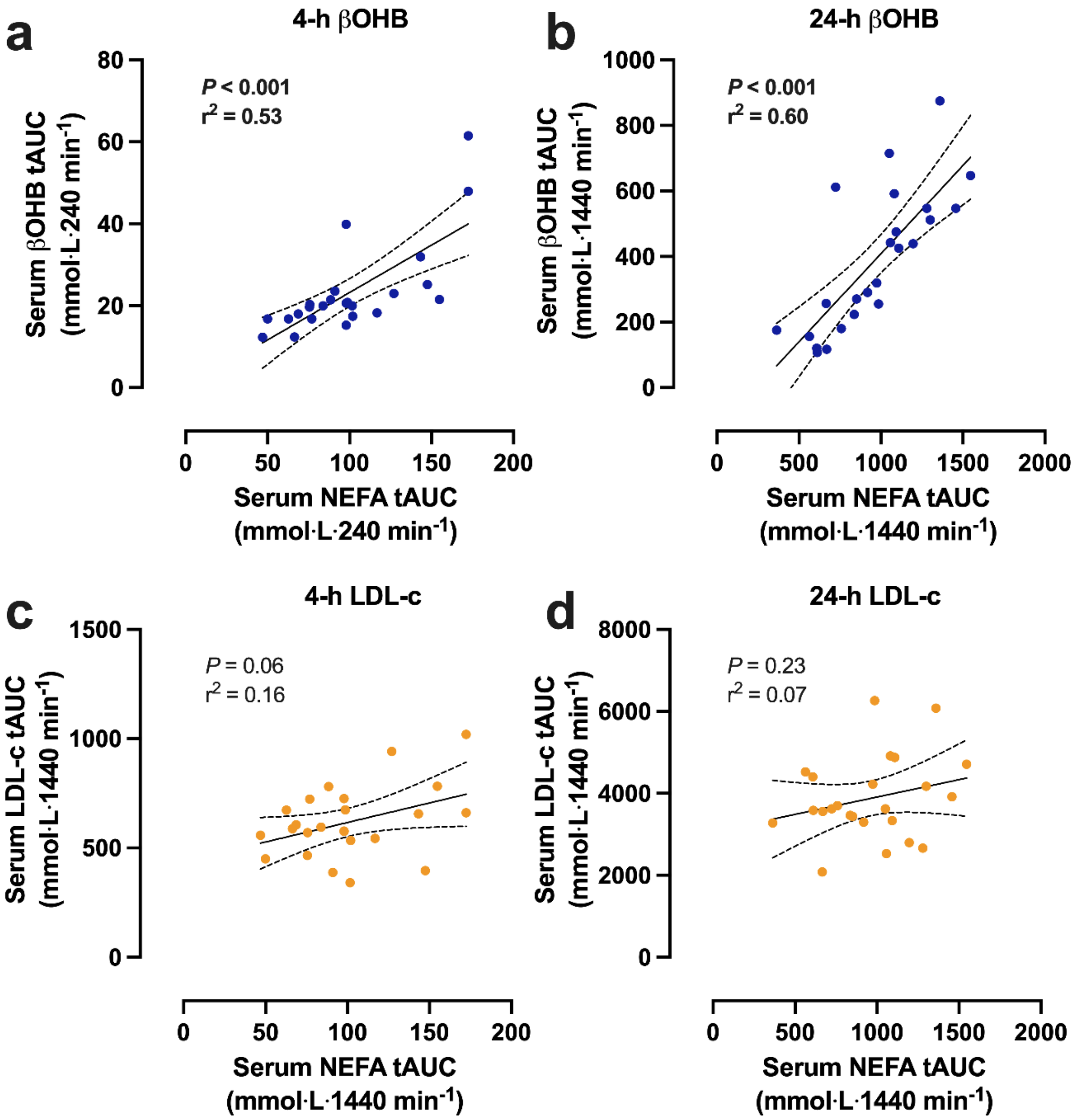


**Supplemental Figure 4.** Pearson correlation coefficients between serum non-esterified fatty acid (NEFA) total area under the curve (tAUC) and β-hydroxybutyrate tAUC across 4 hours (a) and 24 hours (b) of a low carbohydrate diet. Correlations between serum NEFA tAUC and LDL-cholesterol tAUC across 4 hours (c) and 24 hours (d) of a low carbohydrate diet. $n = 24$.
